# Smartphone App–Delivered Mindfulness-Based Intervention for Concussion in Adolescents (MBI-4-mTBI): Feasibility Randomized Controlled Trial

**DOI:** 10.2196/84623

**Published:** 2026-05-11

**Authors:** Veronik Sicard, Molly Cairncross, Roger Zemek, Noah D Silverberg, Gary S Goldfield, Nick Reed, Bechara J Saab, Andra Smith, Andrée-Anne Ledoux

**Affiliations:** 1 Children's Hospital of Eastern Ontario Research Institute, 401 Smyth Road, Ottawa, ON, K1H 5B2, Canada, 1 613-737-7600, 1 613-738-4804; 2 Department of Psychology, Simon Fraser University, Burnaby, BC, Canada; 3 BC Children’s Hospital Research Institute, Vancouver, BC, Canada; 4 Rehabilitation Research Program, Centre for Aging SMART, Vancouver Coastal Health Research Institute, Vancouver, BC, Canada; 5 Department of Pediatrics, University of Ottawa, Ottawa, ON, Canada; 6 Department of Psychology, University of British Columbia, Vancouver, BC, Canada; 7 Djavad Mowafaghian Centre for Brain Health, Vancouver, BC, Canada; 8 School of Psychology, University of Ottawa, Ottawa, ON, Canada; 9 Department of Occupational Science & Occupational Therapy, University of Toronto, Toronto, ON, Canada; 10 Mobio Interactive, Singapore, Singapore; 11 Department of Cellular and Molecular Medicine, University of Ottawa, Ottawa, ON, Canada; 12 University of Ottawa Brain and Mind Research Institute, University of Ottawa, Ottawa, ON, Canada

**Keywords:** pediatric, concussion, mindfulness, clinical trial, psychological intervention, youth, digital therapeutics, eHealth, mHealth, mild traumatic brain injury

## Abstract

**Background:**

Persisting symptoms affect about one-third of youth following concussion. Mental health history, distress, and coping style are key predictors of prolonged recovery. Early and scalable psychological interventions, such as mindfulness-based intervention (MBI) delivered via smartphones, may improve patients’ ability to regulate their emotions and neurophysiologically recover, reducing overall symptom burden. However, no digital therapeutic (DTx) trials in adolescents experiencing concussion exist.

**Objective:**

This study primarily aimed to assess the feasibility of conducting a larger randomized controlled trial (RCT) evaluating the effectiveness of a DTx-MBI in adolescents with a concussion compared with an attention-matched sham intervention.

**Methods:**

This was a Health Canada-regulated, parallel-group, blinded, single-crossover feasibility RCT. Adolescents aged 12 to <18 years presenting to a Pediatric Emergency Department or interdisciplinary concussion clinic within 7 days of a physician-diagnosed concussion were approached for participation from November 2022 to June 2024. After providing consent, participants were randomized (1:1), stratified by sex, to either the experimental group (DTx-MBI) or the control group (sham, attention-matched math puzzle game). The DTx-MBI was delivered via the AmDTx platform (Mobio Interactive Pte Ltd, Singapore) as a custom-designed 4-to-8-week program of 8 standardized modules for adolescents with concussion, including audio-recorded guided mindfulness exercises, goal setting, journaling, and psychoeducation. The control intervention, delivered through the same interface, excluded mindfulness content and instead featured the open-source game “2048”. Participants in both groups were encouraged to engage with the app for at least 10 minutes/day, at least 4 days/week. Feasibility criteria to support progression to a full-scale RCT included: eligibility rate >40% of those screened; recruitment rate >50% of eligible participants randomized; intervention credibility >70% scoring above the midpoint on the credibility and expectancy questionnaire (CEQ) at 1 week; retention >75% of randomized participants completing 4-week outcomes; and adherence >70% completing 10 minutes of intervention on at least 4 days/week for 4 weeks.

**Results:**

A total of 124 out of 195 (63.6%) screened youth met eligibility criteria. Of these, 99/124 (79.8%) consented and were randomized to either the DTx-MBI group (n=49, median [IQR] age=15.28 [13.66‐16.19] years, 30 [61.2%] female) or the Sham group (n=50, median [IQR] age=14.92 [13.32‐16.71] years, 30 [60.0%] female). Credibility was high, with 62/83 (74.7%) of participants scoring above the credibility midpoint (DTx-MBI: 75.0%; Sham: 74.4%). Retention was strong, with 89/99 (89.9%) of participants completing the 4-week outcomes (DTx-MBI: 89.8%; control: 90.0%). Overall adherence was moderate (54/99 [54.5%]; DTx-MBI: 59.2%; control: 50.0%), and a little higher among outcome assessment completers (53/89 [59.6%]; DTx-MBI: 63.6%; Sham: 55.6%). Feasibility indicators were similar between groups.

**Conclusions:**

This feasibility trial supports the implementation of a larger RCT, with modifications to enhance adherence, to rigorously evaluate the clinical efficacy of the DTx-MBI. By targeting modifiable psychological risk factors through a scalable digital platform, DTx-MBI could be a low-burden, cost-effective adjunct to pediatric concussion care.

## Introduction

Approximately one-in-three children sustaining a concussion develop persisting symptoms after concussion (PSAC), defined as ongoing physical, cognitive, emotional, and sleep symptoms that persist beyond 4 weeks postinjury [1], and may persist for months [2,3]. PSAC can disrupt academic performance, reduce social engagement, and increase susceptibility to substance use, depression, anxiety, and suicidal ideation [4-7]. Youth recovering from a concussion face an elevated risk of mental health challenges, including a 40% increased likelihood of developing psychiatric disorders, a 47% higher risk of hospitalization for psychiatric conditions, and a 49% increased risk of self-harm behaviors compared with age- and sex-matched youth with an orthopedic injury [4]. Both injury-related and non-injury-related factors contribute to PSAC [3,8,9]; however, the influence of injury-specific factors tends to diminish over time. In contrast, premorbid factors, such as somatic symptoms [10], migraines [3], cognitive difficulties [11], attention and mood disorders [12], anxiety [13], and maladaptive coping strategies [13], remain strong predictors of prolonged recovery [10,14]. Early interventions that bolster psychological resilience and emotional regulation may be critical in mitigating symptom burden and preventing chronicity [11,14].

Mindfulness-based interventions (MBIs) offer a promising approach by promoting present-focused practices that cultivate nonjudgmental awareness of thoughts and emotions, thereby improving affect regulation and reducing psychological distress [15]. MBIs have been shown to enhance attention, cognitive flexibility, emotional well-being, and academic performance in children, while reducing anxiety, depressive symptoms, affective reactivity, and fear [16]. In addition, MBIs have been shown to reduce default mode network (DMN) hyperactivity across clinical and pediatric populations [17-22], and since concussion similarly involves DMN disruptions [23-27], MBIs may confer neurophysiological benefits postconcussion. MBIs may target several known preinjury risk factors for PSAC, including mood disorders [28-30], somatization [28,31,32], headache disorders [33], and chronic pain [34]. MBIs may improve fatigue, depression, self-efficacy, and quality of life in individuals of different ages with persistent symptoms following mild-to-moderate traumatic brain injuries, including concussions [35-38]. However, these in-person programs are often costly, time-intensive, and not always accessible [35-38]. Digital therapeutics (DTx) have emerged as scalable tools for delivering psychological interventions. Evidence supports the effectiveness of brief web-based MBIs in reducing anxiety, perceived stress, and negative mood across different populations [39-43]. Moreover, a recent meta-analysis found that app-based MBIs show small to moderate effectiveness in improving stress, mood, and quality of life among users without a recent concussion [44]. However, the use of short, app-based DTx-MBIs in pediatric concussion remains unexplored.

This study aimed to assess the feasibility of conducting a larger randomized controlled trial (RCT) evaluating the effectiveness of DTx-MBI to reduce symptom burden and reduce risk of PSAC in youth with a concussion compared with an attention-matched sham intervention by examining recruitment efficiency, intervention credibility, intervention adherence, and participant retention rates at week 4 postenrollment. The secondary research objectives included the following. (1) Determine the participants’ treatment expectations and satisfaction with both interventions. (2) Explore potential efficacy signals across clinical outcomes (ie, concussion symptoms, quality of life, fatigue, anxiety, depression, resiliency, self-efficacy, mindfulness, and cognitive function) to inform future sample size calculations. (3) Examine safety by comparing adverse events between interventions. (4) Assess the feasibility of incorporating a neuroimaging component into the larger trial by examining recruitment and retention for the neuroimaging subset, as well as intervention credibility and intervention adherence among participants who completed neuroimaging (Multimedia Appendix 1 [26,45]). (5) Explore differences in baseline characteristics, adherence, and attrition rates between the experimental group and control group. Experimental group: participants who decided to complete the study at 4 weeks and participants who continued for an additional 4 weeks of MBI for weeks 5‐8; Control group: participants who decided to complete the study at 4 weeks and who crossed over to the experimental intervention for weeks 5‐8 (Multimedia Appendix 1 [26,45]).

## Methods

### Study Design

**Figure 1.**
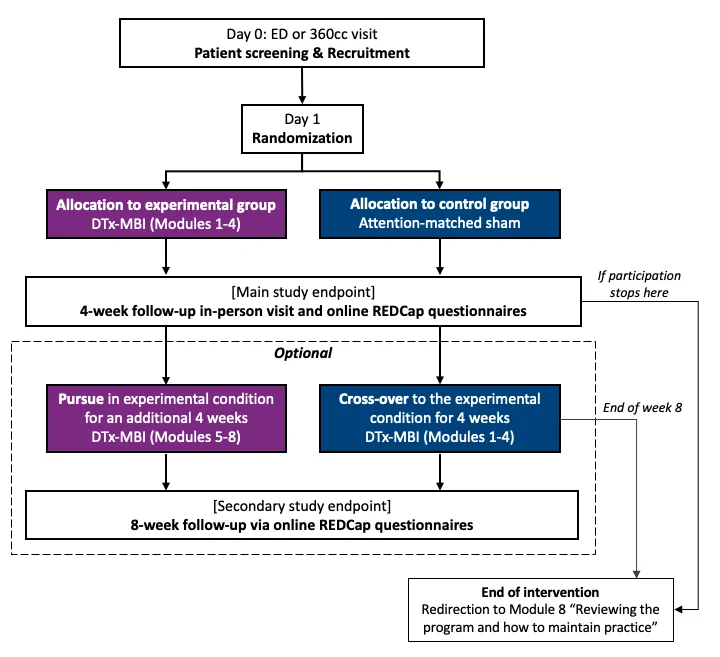
Graphical depiction of study design. 360cc: 360 Concussion Care; DTx-MBI: digital therapeutics mindfulness-based intervention; ED: emergency department; REDCap: Research Electronic Data Capture.

This Health Canada-regulated feasibility RCT (ClinicalTrials.gov: NCT05105802) used a parallel-group (1:1), blinded, single-crossover design. Participants in the control group who completed the initial 4-week study and wished to pursue the experimental intervention crossed over (modules 1‐4) during weeks 5‐8, while those in the experimental group could extend their MBI training for an additional 4 weeks (modules 5‐8; Figure 1). To maintain focus on the primary endpoints, only the 4-week outcomes are presented in the main text; week-8 outcomes are reported in Multimedia Appendix 1 [26,45]. Methods and results of the feasibility of the magnetic resonance imaging (MRI) component are presented in Multimedia Appendix 1 [26,45].

### Ethical Considerations

This study was approved by the Research Ethics Board (protocol #20/720X) of the Children’s Hospital of Eastern Ontario (CHEO) Research Institute in August 2020. Authorization from Health Canada was granted in July 2022. The study was carried out according to the principles outlined in the Declaration of Helsinki and Good Clinical Practices, and within the laws and regulations of the Tri-Council Policy Statement.

Before consent was sought, the study was introduced, and its purpose was explained verbally. The consent form also presented the purpose of the research, the procedures, potential risks and benefits, and the use and security of the data. Informed written consent was obtained from the parents, and participants were deemed capable of consenting. Informed assent was obtained from the participants deemed by the research assistant (RA) to be cognitively unable to provide informed consent.

The participants were remunerated with a CAD $20 (US $15) gift certificate (eg, Tango) for completing surveys. Participants received an additional CAD $20 (US $15) gift certificate for completing the MRIs. The gift certificate was sent electronically to their email address. They also received a parking voucher or vouchers for in-person meetings and a letter attesting that they have completed 20 hours of volunteer work (5 hours per week completing the intervention).

Participant information was coded using study identification numbers. Research personnel took all appropriate and customary steps to ensure that data remain secure and that patient privacy and confidentiality are maintained.

### Procedures

The full protocol was published previously [46]. Briefly, participants were recruited from the CHEO emergency department (ED) and the 360 Concussion Care (360cc) clinic, an interdisciplinary tertiary health clinic in Ottawa, from October 2022 to June 2024. Families of adolescents with a concussion were approached by an RA to discuss the research study. Interested families completed a screening form on a tablet with the RA, and if eligible (concussion confirmed by a physician) and enrolled, filled out electronic questionnaires via Research Electronic Data Capture (REDCap) [47 48] to collect patient demographics, diagnostic history, injury characteristics, and a concussion symptoms checklist.

The day after the ED or 360cc visit, an RA called participants to randomize them into either the experimental or control group. They provided instructions for installing AmDTx on the participant’s mobile device of their choice. Participants completed Day 1 surveys by phone or via REDCap and began the intervention that same day. At the 4-week mark, participants attended an in-person appointment to complete the cognitive testing at either the CHEO Research Institute or the Royal Ottawa Hospital (for those enrolled in the neuroimaging component; see below). After this visit, participants in the experimental group were offered 4 additional weeks of psychoeducation modules and mindfulness practices. Control group participants were given the option to cross over and begin the DTx-MBI. Follow-up questionnaires were completed on REDCap at 1, 2, 4, and 8 weeks. A subset from each group was prospectively approached and enrolled for a neuroimaging (Multimedia Appendix 1 [26,45]).

### Randomization, allocation concealment, and blinding

Participants were randomly assigned to one of 2 groups using a 1:1 allocation ratio stratified by sex. (1) Experimental group: DTx-MBI program, or (2) control group: sham digital attention-matched puzzle game. The CHEO Clinical Research Unit provided data management services for this study and retained randomization codes. A statistician who was not involved in the study created and maintained the master list.

Only the RAs responsible for randomization and app setup were unblinded to participants’ group assignments, as this was necessary for intervention setup and delivery. These RAs were blinded to the randomization sequence until the point of group assignment and were not involved in outcome assessment or data analysis.

During the consent process and follow-up, we gave controlled information to participants about both interventions, while keeping them unaware of the hypothesis, design, and treatment assignment (experimental or control). The study hypothesis and the distinction between experimental and control conditions were disclosed only at the 4-week follow-up, at which point DTx-MBI participants could choose to extend their intervention and the Sham participants could cross over to the experimental condition.

### Participants

Adolescents presenting to the ED or 360cc within 7 days of sustaining a direct or indirect traumatic brain injury were eligible to participate if they: (1) were aged 12 to <18 years; (2) had a diagnosed concussion (by a physician); (3) had 1 highest level of certainty symptom (eg, loss of consciousness, posttraumatic amnesia) or 2 higher level of certainty symptoms (eg, nausea/vomiting, headache) immediately or within 1 hour of injury from the adapted version of the Centers for Disease Control and Prevention tiered framework [49]; (4) had a score of >4 on the Predicting and Preventing Postconcussive Problems in Pediatrics clinical risk score, a validated prediction score to identify those at risk of poorer outcomes at 4 weeks postinjury [3]; (5) were proficient in English.

Patients were excluded if they presented with traumatic brain injuries with any of the following: (1) Glasgow Coma Scale score of ≤13; (2) trauma-related abnormality on standard neuroimaging studies (if performed) [50]; (3) neurosurgical operative intervention, intubation, or intensive care required; (4) multisystem injuries with the treatment requiring hospital admission, operating room, or procedural sedation in ED; (5) severe neurological developmental delay resulting in communication difficulties; (6) intoxication to alcohol or drugs at the time of ED/360cc presentation as per clinician judgment; (7) no clear history of head trauma as the primary event to the concussion (eg, seizure and migraine attack); (8) prior psychiatric hospitalization; (9) inability to obtain written informed consent or assent; (10) legal guardian not present (certain forms needed to be completed by parents or legal guardians); (11) no internet and mobile or tablet access; and (12) was in psychological therapy at enrollment.

### Intervention

#### Experimental Group: DTx-MBI

The DTx-MBI program was delivered via the AmDTx platform and consisted of a 4-to-8-week, custom-made program (containing 8 psychoeducation modules) for adolescents with concussions, including goal setting, check-ins on mood and stress, audio-recorded psychoeducation, guided meditations, and journaling. Each standardized module was unlocked as the participant progressed through the program. Participants were encouraged to participate in the DTx-MBI activities for at least 10 minutes every day, for a minimum of 4 days/week over the study period. Those who chose to end their participation at the end of week 4 were immediately directed to module 8, “Reviewing the program and how to maintain practice,” while those who decided to continue moved on to modules 5 through 8. The DTx had previously undergone pilot testing demonstrating acceptability, credibility, and usability [51].

#### Control Group: Attention-Matched Sham Comparator

The sham digital math puzzle was delivered on the same main interface within AmDTx*,* including the Snapshot (see below); however, these participants did not have access to any of the MBI training or psychoeducational content. The sham DTx was the open-source math game called “2048”, developed by Gabriele Cirulli [52]. The game required participants to slide numbered tiles on a grid and combine matching numbers, aiming to reach 2048 by creating larger numbers through merges. Each move adds a new tile, and the game ends when the grid is full with no possible merges. The game is programmed to only be accessible for 10 minutes per session. The selected control task has precedent as a digital comparator and has shown no effects on stress, psychological well-being, or symptoms in prior studies [42,53].

#### Snapshot

AmDTx includes 4 simple tools for tracking patient well-being over time, collectively called the Snapshot, in which participants in both groups were prompted to complete before and after each session:(1) 30-second “selfie*”* video analyzed via photoplethysmography [54] and a deep neural network to quantify psychological stress from heart rate [55] (2) a digital emotion mapping board (circumplex) to capture emotional stress levels; (3) a subjective stress slider, benchmarked against standard psychological surveys [42]; and (4) an open-text personal notes field. The snapshot feature qualified as meaningful engagement; however, completing the snapshot alone did not fulfill the requirement.

#### Standard of Care

All participants received standard-of-care [56] instruction pamphlets provided by RAs in the ED or at 360cc. The pamphlet includes advice to refrain from physical and cognitive exertion for the first 24 to 48 hours after injury. After the initial rest period, low-to-moderate levels of physical and mental activity can be started, including screen time, provided that the activities do not cause a recurrence or exacerbation of symptoms. It further advises that adolescents avoid activities with an increased risk of reinjury until fully asymptomatic and cleared by their primary care or other medical providers. The study intervention was therefore intended as a supplement to usual care rather than a replacement for standard concussion management.

### Measures and Outcomes

#### Primary Outcomes

The prespecified feasibility targets for progressing to a definitive trial were informed by established benchmarks from previous feasibility trials [57,58] and our experience recruiting pediatric concussion patients from the ED [59]. We used a traffic light system to assess the feasibility of proceeding to a full-scale trial [60]. Progression is classified as feasible without modifications (green), feasible with adjustments (amber), or infeasible due to unresolvable issues (red). Advancement to a full-scale trial is determined by the criterion with the least favorable performance. The 5 prespecified targets are outlined in Table 1. Credibility was assessed using the CEQ (questions 1‐3) at week 1 postenrollment [61], and retention was defined as completing the health behavior inventory at the 4-week follow-up [62].

**Table 1.** Feasibility benchmarks and performance.

Feasibility outcomes	Prespecified benchmark		
	Green zone (go)	Amber zone (amend)	Red zone (stop)
Eligibility	>40% of patients screened are eligible	20%‐40% of patients screened are eligible	<20% of patients screened are eligible
Recruitment	>50% of eligible patients are randomized	30%‐50% of eligible patients are randomized	<30% of eligible patients are randomized
Credibility	>70% of participants score above the scale mid-point^a^	50%‐70% of participants score above the scale mid-point^a^	<50% of participants score above the scale mid-point^a^
Retention	>75% of participants complete the follow-up assessment at 4-week^b^	50%‐75% of participants complete the follow-up assessment at 4-week^b^	<50% of participants complete the follow-up assessment at 4-week^b^
Adherence	>70% of participants complete the minimal requirement (ie, 10 minutes of activity, 4 times/week)	50%‐70% of participants complete the minimal requirement, that is, approximately (ie, 10 minutes of activity, 4 times /week)	<50% of participants who completed the outcomes complete the minimal requirement (ie, 10 minutes of activity, 4 times/week)

a Credibility and expectancy questionnaire questions 1 to 3.

b Health behavior inventory.

#### Secondary Outcomes

Treatment expectancy was assessed with the CEQ (questions 4 to 6) at week 1 after enrollment [61]. Expectancy was considered good if >70% participants scored above the midpoint.

Intervention satisfaction was assessed with the modified version (to align with the digital nature of the intervention) of the Client Satisfaction Questionnaire at 4 weeks [63]. The intervention was deemed satisfactory if >70% of participants scored above the midpoint on the questionnaire.

Safety of the MBI was determined by capturing and monitoring the worsening of symptoms and adverse events associated with the app or intervention. Adverse events were defined as any unscheduled visits to the ED or primary medical providers because of exacerbation of symptoms during the study participation. Events were considered intervention-related if they involved symptom provocation during or within 30 minutes of DTx use. The determination of whether an adverse event was related to the intervention was made by the Qualified Investigator, based on predefined criteria established by the Data and Safety Monitoring Board and reviewed by the institutional quality assurance and regulatory compliance personnel. Participants were asked about possible adverse events at the 1-, 2-, 4-, and 8-week follow-ups.

Other outcome measures were collected at baseline (in the ED or on day 1 for 360cc) and at 4- and 8-week follow-ups, unless otherwise specified:

- Symptom burden: Health behavior inventory, assessed retrospectively for preinjury and acute postinjury symptoms, with additional assessment at 1 and 2 weeks follow-ups [62];
- Quality of life: Pediatric Quality of Life Inventory (PedsQL, version 4.0) [64,65];
- Fatigue: PedsQL Multidimensional Fatigue Scale (PedsQL-MFS, version 4.0) [66,67];
- Depression symptoms: Center for Epidemiologic Studies Depression Scale for Children [68];
- Anxiety: Severity Measure for Generalized Anxiety Disorder–Child Age 11‐17 (DSM-5 GAD-C) [69];
- Resilience: Connor-Davidson Resilience Scale-10 [70];
- Mindfulness: Child and Adolescent Mindfulness Measure [71,72];
- Self-efficacy: Self-Efficacy Questionnaire for Children [73];
- Cognition: National Institutes of Health Toolbox (NIH Toolbox) [74,75] assessed fluid cognition on week 4 only: Fluid Cognition composite score, and Picture Sequence Memory, List Sorting Working Memory, Flanker Inhibitory Control and Attention, Dimensional Change Card Sorting, and Pattern Comparison Processing Speed tests [76].

#### Adherence

App adherence was quantified based on meaningful in-app activity time, defined as active engagement with core features assigned to each group. For the DTx-MBI group, this included guided meditation exercises, psychoeducational content, and snapshots. For the Sham group, the core features were the “2048” game and snapshots, which served as the control activities for engagement tracking. Any single app session lasting <3 minutes were excluded from adherence calculations, as this duration was deemed insufficient to complete meaningful activities. Weekly adherence was calculated by summing the total seconds of the qualifying app session within every 7 days over the 4-week study period.

### Statistical Analysis

#### Overview

Descriptive statistics were used to summarize participant characteristics, with medians reported for continuous variables and frequencies for categorical variables. Feasibility outcomes were reported as proportions. For outcome measures at 4 and 8 weeks, medians and IQRs were calculated for each group. In a post hoc exploratory analysis, a multiple regression was conducted to examine factors associated with app engagement (in seconds, square root transformed to address nonnormality and improve model assumptions) over the 4-week intervention period. The regression model included group allocation, age, sex, and preinjury ratings of anxiety, depression, mindfulness, and self-efficacy. All statistical analyses were conducted on R (version 4.4.1; R Core Team, 2024) [77].

#### Sample Size Calculation

Detailed sample size justifications can be found in the published protocol [46]. Briefly, sample size estimates were based on feasibility trial guidelines using the traffic light system [60,78]. To achieve >90% power for each of the 5 feasibility criteria, the highest requirement was adherence (n=55). Accounting for a 20% attrition, 70 participants were needed. Assuming 40% eligibility and 50% recruitment rates, ≈350 children needed to be screened.

## Results

### Participants

**Figure 2.**
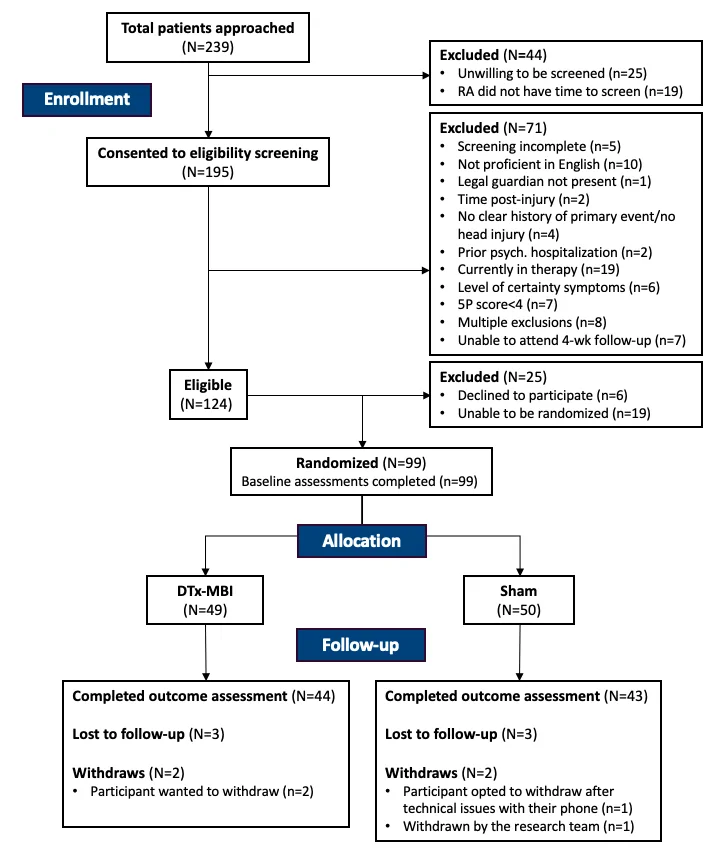
Participant flow diagram. 5P: Predicting and Preventing Postconcussive Problems in Pediatrics study clinical risk score; DTx-MBI: digital therapeutics mindfulness-based intervention; RA: research assistant.

A total of 99 out of 124 eligible patients were randomized to DTx-MBI (n=49; median age [IQR]=15.28 [13.66‐16.19] years; 30 [61%] female) or Sham groups (n=50; median age [IQR]=14.92 [13.32‐16.71] years; 30 [60%] female) (Figure 2). A total of 65 participants were recruited from the ED and 34 from 360cc. Demographics and clinical characteristics were comparable between groups among all randomized participants and those who completed the 4-week follow-up (Table 2).

**Table 2.** Participants’ demographic and clinical characteristics.

Variables	Randomized		Completed the 4-week intervention	
	DTx-MBI^a^ (n=49)	Sham (n=50)	DTx-MBI (n=44)	Sham (n=45)
Demographics				
Age (years), median (IQR)	15.28 (13.66‐16.19)	14.92 (13.32‐16.71)	14.92 (13.61‐16.17)	14.84 (13.30‐16.63)
Female sex, n (%)	30 (61.2)	30 (60.0)	27 (61.4)	26 (57.8)
Diagnostic history				
Previous concussion, n (%)	21 (42.9)	23 (46.0)	20 (45.5)	21 (46.7)
Number of previous concussions, median (IQR)	1 (1-2)	2 (1-2)	1 (1-2.25)	2 (1-2)
Migraines, n (%)	4 (8.2)	2 (4.0)	4 (9.1)	2 (4.4)
Learning disabilities, n (%)	4 (8.3)^b^	11 (22.0)	4 (9.3)^b^	9 (20.0)
Attention-deficit/hyperactivity disorder (ADHD), n (%)	7 (14.6)^b^	6 (12.0)	7 (16.3)^b^	4 (8.9)
Other developmental disorders, n (%)	1^c^ (2.1)^b^	1^d^ (2.0)^b^	1^c^ (2.3)^b^	0 (0)
Anxiety, n (%)	5 (10.4)^b^	9 (18.0)	5 (11.6)^b^	8 (17.8)
Depression, n (%)	5 (10.4)^b^	1 (2.0)	4 (9.3)^b^	1 (2.2)
Sleep disorders, n (%)	1 (2.1)^b^	2 (4.0)	1 (2.3)^b^	0 (0)
Other mental health diagnoses, n (%)	1^e^ (2.1)^b^	0 (0)	1^e^ (2.3)^b^	0 (0)
Clinical variables				
Recruitment site, n (%)				
Emergency department	30 (61.2)	35 (70.0)	27 (61.4)	30 (66.7)
360 Concussion Care	19 (38.8)	15 (30.0)	17 (38.6)	15 (33.3)
Hours post-injury at enrollment, median (IQR)	28.83 (3.23‐99.27)	28.69 (7.82‐96.11)	30.50 (4.39‐96.82)	35.05 (8.63‐108.17)
5P risk score^f^, median (IQR)	7.00 (6.00‐8.00)	7.00 (6.00‐8.00)	7.00 (6.00‐8.00)	7.00 (6.00‐8.00)
Low risk, n (%)	0 (0)	1 (2.0)	0 (0)	1 (2.2)
Medium risk, n (%)	38 (77.6)	37 (74.0)	34 (77.3)	33 (73.3)
High risk, n (%)	11 (22.4)	12 (24.0)	10 (22.7)	11 (24.4)
Loss of consciousness, n (%)	9 (18.8)^b^	5 (10.2)^b^	8 (18.6)^b^	5 (11.1)
Amnesia, n (%)	16 (33.3)^b^	16 (32.0)	15 (34.1)^b^	14 (40.0)
Previous experience with mindfulness training therapy, n (%)	3 (6.3)^b^	4 (8.0)	2 (4.7)^b^	2 (4.4)
Prior experience with mindfulness, n (%)^g^	7 (14.6)^b^	7 (14.0)	5 (11.6)^b^	6 (13.3)
Mechanism of injury, n (%)				
Fall down stairs	1 (2.0)	1 (2.0)	1 (2.3)	1 (2.2)
Sports	37 (75.5)	34 (68.0)	32 (72.7)	30 (66.7)
Fall from standing, walking, or running	3 (6.1)	5 (10.0)	3 (6.8)	5 (11.1)
Ran into stationary object	0 (0)	2 (4.0)	0 (0)	2 (4.4)
Other mechanism	7 (14.3)	7 (14.0)	7 (15.9)	6 (13.3)
Missing	1 (2.0)	0 (0.0)	1 (2.3)	1 (2.2)
Retrospective HBI^h^, median (IQR)	10.50 (4.00‐17.00)^b^	11.00 (5.25‐17.75)	11.00 (4.00‐17.00)^b^	11.00 (4.00‐16.00)

a DTx-MBI: digital therapeutics mindfulness-based intervention.

b Missing values (mv)=1.

c Word retrieval/language delay.

d Autism spectrum.

e Unspecified.

f Low risk=0‐3, Medium risk=4‐8, Higher risk=9‐12.

g Originally coded as “None,” “A little”, “Some”, “A lot” but recoded as “Yes” or “No.”

h HBI: health behavior inventory.

### Primary Analyses: Feasibility Outcomes at 4-Week Postenrollment

Four out of the 5 prespecified feasibility outcomes met the “green zone” benchmarks.

#### Eligibility

A total of 124 out of 195 (63.6%) participants were screened, exceeding the >40% eligibility criteria.

#### Recruitment

A total of 99 out of 124 (80%) eligible participants consented and were randomized, surpassing the 50% benchmark.

#### Credibility

A total of 62 out of 83 (75%) of participants scored above the scale midpoint, with similar results between the DTx-MBI (33/44, 75%) and Sham groups (29/39, 74%), exceeding the 70% target.

#### Retention

A total of 89 out of 99 (90%) of participants completed the 4-week follow-up assessments, with similar completion rates between the DTx-MBI (44/49, 90%) and Sham groups (45/50, 90%), meeting the 75% benchmark.

#### Adherence

A total of 53 out of 89 (60%) of participants completed the minimal requirement of activity, below the 70% target, falling into the amber zone. The DTx-MBI group showed slightly higher adherence (28/44, 64%) compared with the Sham group (25/45, 56%).

### Secondary Analyses

#### Treatment Expectancy and Satisfaction

A total of 61 out of 83 (74%) participants scored above the scale mid-point on treatment expectancy (DTx-MBI: 34/44, 77%; Sham: 27/39, 69%). Moreover, intervention satisfaction was good, with 71/86 (83%) participants scoring above the scale mid-point (DTx-MBI: 37/43, 86%; Sham: 34/43, 79%).

#### Efficacy Signal

Given the nonnormal distribution of the outcome measures, medians and IQRs were calculated for all outcomes at baseline and week 4 (Table 3) and week 8 (Tables S1 and S2 in Multimedia Appendix 1 [26,45]). These analyses were intended to inform future sample size calculations; the study was not powered to detect statistically significant between-group differences.

**Table 3.** Efficacy signal for outcomes at week 4 postenrollment.

Outcomes	Baseline (preintervention)		4-week endpoint	
	DTx-MBI^a^ (n=44)^b^	Sham (n=45)^b^	DTx-MBI (n=44)	Sham (n=45)
Retrospective HBI^c^, median (IQR)	11.00 (4.00‐17.00)^d^	11.00 (4.00‐16.00)	—^e^	—
HBI (symptom burden), median (IQR)	26.00 (17.00‐34.50)	25.00 (13.00‐32.00)	16.50 (10.00‐23.00)	13.00 (6.50‐22.50)
PSAC,^f^ n (%)	—	—	5 (11.6)^d^	5 (11.6)
PedsQL^g^ (quality of life), median (IQR)	80.00 (71.72‐90.00)^d^	80.63 (70.43‐89.88)^d^	81.17 (67.93‐89.92)	84.38 (70.16‐92.11)
Physical	90.63 (76.56‐96.88)^d^	87.50 (80.47‐93.75)	90.63 (71.09‐93.75)	90.63 (71.88‐96.88)
Emotional	75.00 (65.00‐95.00)^d^	75.00 (58.75‐90.00)	77.50 (60.00‐95.00)	75.00 (65.00‐92.50)
Social	95.00 (75.00‐100.00)^d^	90.00 (73.75‐100.00)^d^	95.00 (73.75‐100.00)	95.00 (85.00‐100.00)
School	70.00 (60.00‐85.00)^d^	70.00 (55.00‐86.25)^d^	67.50 (53.75‐80.00)	75.00 (52.50‐90.00)
PedsQL-MFS^h^ (fatigue), median (IQR)	70.83 (61.11‐80.56)^d^	69.44 (62.15‐80.90)^d^	66.67 (54.51‐80.90)	73.61 (52.08‐86.11)
Cognitive	70.84 (52.08‐77.08)^d^	77.08 (58.33‐87.50)^d^	68.75 (50.00‐71.88)	70.83 (52.08‐86.11)
Sleep/Rest	62.50 (52.08‐77.08)^d^	62.50 (50.00‐71.88)^d^	58.33 (50.00‐71.88)	66.67 (52.08‐75.00)
General	79.17 (66.67‐87.50)^d^	79.17 (66.67‐91.67)^d^	75.00 (57.29‐91.67)	75.00 (62.50‐91.67)
CES-DC^i^ (depression), median (IQR)	4.00 (3.00‐9.00)	5.00 (3.00‐7.00)	6.00 (3.75‐10.00)	6.00 (4.00‐8.00)
DSM-5 GAD-C^j^ (anxiety), median (IQR)	4.00 (0.50‐9.00)^d^	5.00 (1.00‐10.00)^d^	3.00 (1.00‐10.25)	5.00 (0.00‐9.00)
CD-RISC-10^k^ (resilience), median (IQR)	28.00 (23.50‐30.50)^d^	28.00 (24.75‐31.00)^d^	25.50 (10.75‐30.50)	26.00 (20.00‐32.00)
CAMM^l^ (mindfulness), median (IQR)	32.00 (26.50‐37.00)^d^	31.00 (25.00‐36.00)^d^	33.00 (24.00‐38.00)	32.00 (26.50‐36.50)
SEQ-C^m^ (self-efficacy), median (IQR)	85.00 (73.00‐94.50)^d^	82.50 (75.00‐90.25)^d^	80.50 (67.75‐89.25)	81.00 (70.50‐95.00)
Emotional	28.00 (23.00‐31.50)^d^	27.00 (24.00‐30.00)^d^	26.50 (21.75‐32.00)	26.00 (22.00‐32.50)
Social	29.00 (26.00‐33.00)^d^	29.00 (26.00‐31.25)^d^	27.00 (24.00‐32.00)	29.00 (25.00‐32.00)
Academic	26.00 (22.00‐32.00)^d^	27.00 (24.00‐30.25)^d^	26.00 (22.00‐33.00)	27 (21.50‐32.00)
NIH^n^ toolbox (cognition), median (IQR)				
Fluid cognition composite score (age-corrected)	—	—	103.00 (85.00‐113.00)^o^	104.00 (91.50‐117.00)^p^
Flanker (inhibition control, attention)	—	—	85.00 (75.50‐97.00)^q^	89.00 (84.00‐100.50)^p^
List sorting (working memory)	—	—	98.00 (92.50‐103.50)^r^	100.00 (84.00‐100.50)^p^
Dimensional change card sort (executive functions)	—	—	101.00 (85.50‐115.00)^r^	105.00 (86.00‐120.00)^p^
Pattern comparison (processing speed)	—	—	112.00 (94.50‐128.00)^r^	103.00 (93.50‐124.00)^p^
Picture sequence (memory)	—	—	105.00 (93.00‐110.00)^r^	105 (96.00‐115.00)^p^
Agreed to continue to 8-week, n (%)	—	—	10 (22.7)	11 (25.6)

a DTx-MBI: digital therapeutics mindfulness-based intervention.

b Include participants who completed the 4-week protocol.

c HBI: health behavior inventory.

d Missing value (mv)=1.

e Not applicable.

f Persisting Symptoms After Concussion (PSAC) was determined through reliable change z scores of≥1.65 [79]. The reliable change score compares the parent’s retrospective rating of preinjury total symptoms with the child’s ratings reported at the 4-week follow-up, based on a formula derived from regression analyses in children with orthopedic injuries.

g PedsQL: Pediatric Quality of Life Inventory, version 4.0.

h MFS: Multidimensional Fatigue Scale.

i CES-DC: Center for Epidemiologic Studies Depression Scale for Children.

j DSM-5 GAD-C: Severity Measure for Generalized Anxiety Disorder–Child Age 11‐17.

k CD-RISC-10: Connor-Davidson Resilience Scale-10.

l CAMM: Child and Adolescent Mindfulness Measure.

m SEQ-C: Self-Efficacy Questionnaire for Children.

n NIH: National Institutes of Health.

o Missing value (mv)=11.

p Missing value (mv)=4.

q Missing value (mv)=10.

r Missing value (mv)=9.

#### Safety of the App

Two participants from the DTx-MBI group reported unscheduled medical visits during their study participation, but no visits met our predefined safety criteria for adverse events associated with the use of the application: one participant visited the ED for a second concussion in week 3, while the other visited for worsening of concussion symptoms prior to AmDTx use.

### Post Hoc Exploration: Factors Associated With Activity Engagement

Given low adherence, we conducted a post hoc analysis to examine factors associated with intervention adherence. In the post hoc exploratory multiple regression, none of the demographic or preinjury factors showed statistically significant associations with activity engagement during the 4-week intervention period (Table 4).

**Table 4.** Demographic and preinjury factors associated with activity engagement (measured in seconds) over the 4-week intervention period.

Variables	Estimate	SE	Statistic	*P* value	95% CI
Intercept	59.62	121.06	0.42	.62	−181.49 to 300.73
Group	−0.45	13.84	−0.03	.97	−28.02 to 27.12
Age	1.00	4.47	0.22	.82	−7.91 to 9.91
Sex	21.08	15.90	1.33	.19	−10.60 to 52.75
HBI^a^ (symptoms)	−0.81	0.69	−1.18	.24	−2.18 to −0.56
DSM-5 GAD-C^b^ (anxiety)	−9.72	15.12	−0.64	.52	−39.83 to 20.39
CES-DC^c^ (depression)	1.29	2.31	0.56	.58	−3.32 to 5.89
CAMM^d^ (mindfulness)	−1.55	1.36	−1.14	.26	−4.26 to 1.16
SEQ-C^e^ (self-efficacy)	1.02	0.61	1.68	.10	−0.19 to 2.24

a HBI: health behavior inventory.

b DSM-5 GAD-C: Severity Measure for Generalized Anxiety Disorder–Child Age 11‐17.

c CES-DC: Center for Epidemiologic Studies Depression Scale for Children.

d CAMM: Child and Adolescent Mindfulness Measure.

e SEQ-C: Self-Efficacy Questionnaire for Children.

**Figure 3.**
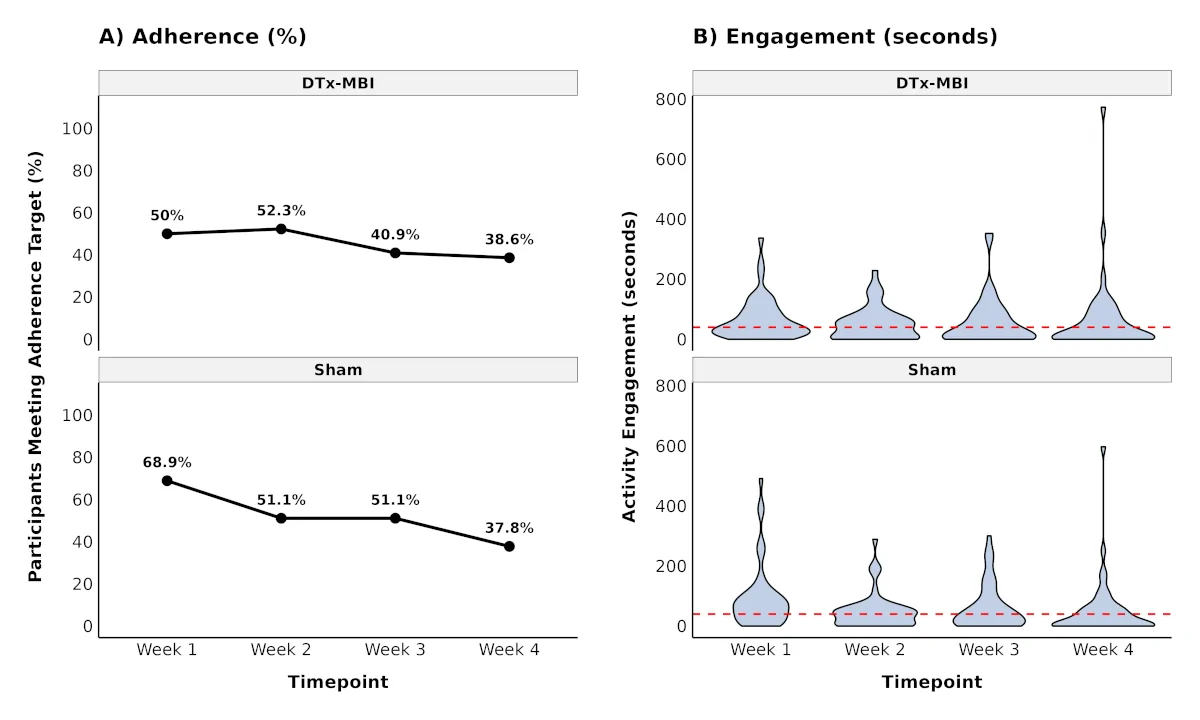
Weekly adherence. (A) Percentage of participants meeting the weekly 40-minute app activity engagement target across the 4 weeks of protocol within the experimental (DTx-MBI) and control (attention-matched cognitive Sham comparison; Sham) groups. Data points show exact percentages for each group at each week. (B) Distribution of weekly app activity engagement (in minutes) shown as violin plots for each group. Each violin plot shows the density of engagement duration, with a larger width indicating higher data concentration. Black dots represent median values, and vertical lines span the IQR (****Q1**-Q3**). The red dashed horizontal line at 40 minutes indicates the target engagement threshold. Only participants who completed the 4-week outcomes were included in the figure. DTx-MBI: digital therapeutics mindfulness-based intervention.

Among participants who completed the intervention, weekly adherence rates (ie, meeting the threshold of ≥10 minutes of “meaningful” app engagement for 4 days over a 7-day week) declined from week 1 (DTx-MBI: 22/44, 50%; Sham: 31/45, 69%) to week 4 (DTx-MBI: 17/44, 39%; Sham: 17/45, 38%). Similarly, median weekly engagement time decreased from 40.9 minutes (IQR 20.2‐89.9) to 17.1 minutes (IQR 0‐76.8) in the DTx-MBI group and from 75.2 minutes (IQR 26.2‐108.1) to 12.6 minutes (IQR 0‐60.0) in the Sham group over the 4 weeks (Figure 3).

## Discussion

### Principal Findings

This feasibility RCT indicated that a larger-scale trial evaluating the effectiveness of DTx-MBI in youth with a concussion compared with an attention-matched sham intervention is viable, with 4 out of the 5 prespecified feasibility benchmarks—eligibility, recruitment, credibility, and retention—being met or exceeded. Only adherence fell slightly below the target threshold, with engagement levels comparable to other digital health interventions in youth [28], suggesting that minor adjustments could improve adherence in the large-scale efficacy RCT.

The high eligibility rate suggests that the inclusion criteria were well aligned with the characteristics of the target population, and the high recruitment rates underscore the strong relevance and suitability of this intervention for the target population. Moreover, retention at 4 weeks approached 90% in both groups, reflecting strong participant commitment and the feasibility of our follow-up strategy. The high credibility and satisfaction across both intervention and control groups support the acceptability of the app-based interventions, regardless of their content. This reinforces findings from our earlier open-label preliminary study [51,80]. In the mixed methods evaluation (n=7), credibility, expectancy, and satisfaction ratings were even higher, although this may have been influenced by the small sample size, open-label design, and participants’ awareness that they would be asked for feedback during the 4-week interview [51]. This blinded RCT strengthens the evidence for the acceptability of the DTx-MBI through a more rigorous randomized design and the inclusion of an attention-matched comparator. Although treatment expectancy was above the scale midpoint for all participants, those in the MBI-DTx reported a qualitatively higher expectancy that the treatment would be helpful, suggesting greater perceived relevance or believability of the experimental intervention. The treatment expectancy ratings in the Sham group were just below the threshold considered “good” in this study, whereas the experimental group met the benchmark. In the efficacy RCT, we will address this potential imbalance by providing participants in the Sham group with additional education about how cognitive training may promote recovery through neuroplasticity during enrollment.

Notably, in this study, no adverse events related to app use were reported, supporting the intervention’s safety. Furthermore, the optional MRI component also met the feasibility benchmarks, with high enrollment and scan completion rates, suggesting that a neuroimaging component can be successfully integrated into future trials.

Adherence, while below the 70% benchmark, remained within an acceptable range (nearly 60%), with slightly higher adherence in the DTx-MBI group across the 4 weeks. Weekly adherence declined over the 4 weeks in both groups, with median weekly engagement time decreasing substantially from week 1 to week 4 in both groups, with a more pronounced decline in the Sham group. This reduction may reflect the natural waning of novelty or motivation over time. This pattern is consistent with Eysenbach’s “Law of Attrition,” which posits that drop-off in usage is an expected and inherent feature of eHealth interventions, as users often disengage over time regardless of intervention quality or intent [81]. The drop-off in app engagement around weeks 2 and 3 may also coincide with the typical course of concussion recovery, a point at which participants may no longer perceive the intervention as necessary [9]. While the DTx-MBI group began with lower adherence rates than the Sham group, they sustained slightly higher engagement by week 4, potentially indicating greater long-term relevance or perceived efficacy or benefit of the mindfulness-based content. Previous studies in adolescents and adults have reported wide variability in adherence to digital MBIs, ranging from 40% to 92% [28]. In this study, we did not identify any participant characteristics associated with lower app engagement. However, prior work suggests that psychological factors such as resilience and coping style may moderate response to behavioral concussion interventions, suggesting that engagement may depend on characteristics not captured here [82]. Future efficacy trials may benefit from enhanced engagement strategies, such as personalized reminders and motivational interviewing. However, such strategies may reduce scalability and accessibility, as they require additional resources and personalization. Implementing them only when early signs of disengagement emerge (as observed in this study around week 2) may help balance these trade-offs.

It is also important to note that the engagement tracking metrics may under-report actual engagement, as the developer’s system was designed to capture only completed “meaningful interactions.” Sessions that were started but not completed (eg, if a participant began a meditation recording but exited before finishing) were not logged. Likewise, activities and assessments (snapshots, journaling, etc) that lasted at least 3 minutes but were not explicitly submitted by the participant once they were done, were not logged. The same goes for the sham app. Based on the developer’s user testing, these incomplete sessions and unsubmitted assessments may account for roughly 10%‐20% of potential meaningful interactions. As such, the engagement data presented here represent only fully completed meaningful interactions, and actual usage may have been modestly higher than reported.

Although not powered for efficacy, both groups seemed to show reductions in symptom burden and improvements in quality of life at the 4-week assessment. The Sham group appeared to report higher sleep and school quality of life at 4 weeks, while these scores declined slightly in the DTx-MBI group. At 8 weeks, among participants who continued with the DTx-MBI, sleep and school quality of life increased, whereas they showed minimal change in the Sham participants who crossed over to the MBI intervention. Moreover, performance on the NIH Toolbox Pattern Comparison task, a measure of processing speed, was modestly higher in the DTx-MBI group compared with the Sham group, although this difference may reflect pre-existing group differences rather than a potential intervention effect since no baseline measures were available for the NIH Toolbox. These descriptive comparisons were not formally tested and are presented only to provide preliminary signals to inform future fully powered trials. Accordingly, these preliminary findings should be interpreted cautiously and warrant further investigation in a fully powered trial to determine the intervention’s efficacy and clarify its impact across recovery outcomes.

### Limitations

Despite overall strong feasibility metrics, this study has some limitations. First, estimates of recruitment, eligibility, adherence, and retention were based on a modest sample size, which may limit precision. Second, the control group’s treatment expectancy was qualitatively lower and just below the benchmark for being considered “good,” which may suggest that some participants suspected they were not receiving the genuine cognitive training intervention, indicating partial unblinding or differential perceived value, despite the use of an active sham. This may have been further compounded by the nature of the control condition, which consisted of math puzzles without concussion-specific content. However, it should be noted that all participants received standard-of-care concussion guidance regardless of group allocation, which is itself expected to support natural recovery and limits the interpretation that any observed differences reflect expectancy alone. These factors will need to be carefully addressed in the design and interpretation of the efficacy trial. Last, as expected for a feasibility study, it was not powered to detect efficacy, and outcome findings should be considered exploratory and interpreted accordingly. This is particularly relevant for weeks 5‐8, which included only a small subset of participants who elected to continue.

### Conclusions

There are a few established preventive nonpharmacological interventions for PSAC in pediatrics [83]; however, psychological approaches are increasingly explored. This feasibility trial supports the implementation of a larger RCT to rigorously evaluate the clinical efficacy of the DTx-MBI in pediatric concussion when compared with an attention-matched cognitive sham delivered on the same digital platform. With refinements to improve adherence, especially in weeks 2 to 4, we will be positioned to evaluate whether this intervention reduces symptom burden and enhances multifaceted recovery outcomes, including quality of life, mood, cognition, and neurophysiology, in youth with a concussion.

## Supplementary material

10.2196/84623 Multimedia Appendix 1 Additional results.

10.2196/84623 Checklist 1 CONSORT-eHEALTH checklist (V 1.6.1).

## Abbreviations

360cc: 360 Concussion Care
CEQ: credibility and expectancy questionnaire
CHEO: Children’s Hospital of Eastern Ontario
DTx: digital therapeutics
ED: emergency department
MBI: mindfulness-based intervention
MRI: magnetic resonance imaging
NIH: National Institutes of Health
PedsQL: Pediatric Quality of Life Inventory
PSAC: persisting symptoms after concussion
RA: research assistant
RCT: randomized controlled trial
REDCap: Research Electronic Data Capture
