# Correction: Smartphone App–Delivered Mindfulness-Based Intervention for Concussion in Adolescents (MBI-4-mTBI): Feasibility Randomized Controlled Trial

**DOI:** 10.2196/110902

**Published:** 2026-11-09

**Authors:** Veronik Sicard, Molly Cairncross, Roger Zemek, Noah Silverberg, Gary S Goldfield, Nick Reed, Bechara J Saab, Andra Smith, Andrée-Anne Ledoux

**Affiliations:** 1 Children's Hospital of Eastern Ontario Research Institute, 401 Smyth Road, Ottawa, ON, K1H 5B2, Canada, 1 737-7600, 1 737-7600; 2 Department of Psychology, Simon Fraser University, Burnaby, BC, Canada; 3 BC Children’s Hospital Research Institute, Vancouver, BC, Canada; 4 Rehabilitation Research Program, Centre for Aging SMART, Vancouver Coastal Health Research Institute, Vancouver, BC, Canada; 5 Department of Pediatrics, University of Ottawa, Ottawa, ON, Canada; 6 Department of Psychology, University of British Columbia, Vancouver, BC, Canada; 7 Djavad Mowafaghian Centre for Brain Health, Vancouver, BC, Canada; 8 School of Psychology, University of Ottawa, Ottawa, ON, Canada; 9 Department of Occupational Science & Occupational Therapy, University of Toronto, Toronto, ON, Canada; 10 Mobio Interactive, Singapore, Singapore; 11 Department of Cellular and Molecular Medicine, University of Ottawa, Ottawa, ON, Canada; 12 University of Ottawa Brain and Mind Research Institute, University of Ottawa, Ottawa, ON, Canada

In “Smartphone App–Delivered Mindfulness-Based Intervention for Concussion in Adolescents (MBI-4-mTBI): Feasibility Randomized Controlled Trial” [1], the authors noted 6 errors.

The outcome measure used to assess anxiety was the Severity Measure for Generalized Anxiety Disorder–Child Age 11‐17 (DSM-5 GAD-C), a validated tool developed alongside the *Diagnostic and Statistical Manual of Mental Disorders* (Fifth Edition; *DSM-5*) for use in pediatric populations, not the Generalized Anxiety Disorder 7-item scale (GAD-7), an adult measure. This affects 5 statements in the article and 1 reference. The corrections below reflect the accurate instrument used throughout data collection and analysis.

First, the following bullet point in the Secondary Outcomes section of the Methods was revised:

*Anxiety: Generalized Anxiety Disorder 7-item scale*

The corrected bullet point now reads:

*Anxiety: Severity Measure for Generalized Anxiety Disorder–Child Age 11‐17 (DSM-5 GAD-C)*

Second, the following row heading in Table 3 was revised:

*GAD-7^j^ (anxiety), median (IQR)*

The row heading now reads:

*DSM-5 GAD-C^j^ (anxiety), median (IQR)*

Third, footnote j in Table 3 was revised from the following:

^*j*^ *GAD-7: Generalized Anxiety Disorder 7-item scale.*

Footnote j now reads:

*^j^DSM-5 GAD-C: Severity Measure for Generalized Anxiety Disorder–Child Age 11‐17.*

Fourth, the following row heading in Table 4 was revised:

*GAD-7^b^ (anxiety)*

The row heading now reads:

*DSM-5 GAD-C^b^ (anxiety)*

Fifth, footnote b of Table 4 was revised from the following:

*^b^GAD-7: Generalized Anxiety Disorder 7-item scale.*

Footnote b now reads:

*^b^DSM-5 GAD-C: Severity Measure for Generalized Anxiety Disorder–Child Age 11‐17.*

Finally, reference 69 was revised from the following:

*69. Spitzer RL, Kroenke K, Williams JBW, Löwe B. A brief measure for assessing generalized anxiety disorder: the GAD-7. Arch Intern Med. May 22, 2006;166(10):1092‐1097. URL: https://jamanetwork.com/* *[doi: 10.1001/archinte.166.10.1092] [Medline: 16717171]*

Reference 69 now reads:

*69. Craske M, Wittchen U, Bogels S, Stein M, Andrews G, Lebeu R. Severity measure for generalized anxiety disorder—child age 11–17. American Psychiatric Association. 2013. URL: https://www.psychiatry.org/getmedia/73e293d0-6355-4258-9cd3-2fc8a5c5b830/APA-DSM5TR-SeverityMeasureForGeneralizedAnxietyDisorderChildAge11To17.pdf [Accessed 2026-08-31]*

The correction will appear in the online version of the paper on the JMIR Publications website together with the publication of this correction notice. Because this was made after submission to PubMed, PubMed Central, and other full-text repositories, the corrected article has also been resubmitted to those repositories.

## Abbreviations

*DSM-5*: *Diagnostic and Statistical Manual of Mental Disorders* (Fifth Edition)
DSM-5 GAD-C: Severity Measure for Generalized Anxiety Disorder–Child Age 11-17
GAD-7: Generalized Anxiety Disorder 7-item scale
