# Supplementary material for: Smartphone App–Delivered Mindfulness-Based Intervention for Concussion in Adolescents (MBI-4-mTBI): Feasibility Randomized Controlled Trial
Source: JMIR Rehabil Assist Technol. 2026 May 11;13:e84623. doi: 10.2196/84623 (PMC13160406; doi:10.2196/84623)
Supplement: Multimedia Appendix 1 [file rehab-v13-e84623-s001.docx]

# **Supplemental Materials. Additional Secondary Results**

**List of Supplemental Materials**

**1) Continuation to week 8**

**Tables**

- **Table S1**. Baseline characteristics, Adherence, and Attrition by Study Completion and Crossover status (i.e., those who stopped study participants at the week 4 endpoint versus those who decided to continue to week 8)
- **Table S2**. Baseline characteristics, adherence, and attrition rates among Sham participants by study completion status (i.e., those who stopped study participation at the week 4 endpoint versus those who decided to crossover to the MBI condition and continue for four weeks)
- **Table S3.** Efficacy signal for outcomes at week 8 post-enrollment

**2) Neuroimaging component**

**Figures**

- **Figure S1**. Participant flow diagram for the neuroimaging component

## **Comparison of Baseline Characteristics, Adherence, and Attrition by Study Completion and Crossover Status**

Given the limited sample size among participants who continued to week 8 (21/87; 24.1% across both groups), quantitative comparisons were not conducted, and findings are presented qualitatively.

10/44 (22.7%) DTx-MBI participants who completed the 4-week study decided to pursue the intervention for an optional, additional 4 weeks. Participants who decided to pursue seemed to qualitatively be enrolled later post-injury, have a higher 5P score, and a higher intervention adherence relative to those who decided to stop their participation at the 4-week endpoint (Table S1).

11/43 (25.6%) Sham participants who completed the 4-week study decided to cross-over to the intervention arm for an optional 4 weeks. Participants who decided to cross-over to the DTx-MBI seemed to qualitatively be enrolled later, have a higher 5P clinical risk score, have a higher incidence of history of concussion and attention deficit-hyperactivity disorder, score higher on baseline quality of life assessment, and have lower adherence intervention relative to those who decided to stop their participation at the 4-week endpoint (Table S2).

**Table S1. Baseline characteristics, among DTx-MBI participants by study completion status (i.e., those who stopped study participation at the week 4 endpoint versus those who decided to continue to week 8)**

| **Variables** | **Study completion at 4-week**  **N=34** | **Continue MBI for an additional 4 weeks ^a^**  **N=10** |
| --- | --- | --- |
| **Demographics** |  |  |
| Age, Median [IQR] | 14.92 [13.66-16.09] | 15.14 [13.74-16.35] |
| Female sex, n (%) | 23 (65.7) | 5 (50.0) |
| **Diagnostic history** |  |  |
| Previous concussion, n (%) | 13 (37.1) | 7 (7.0) |
| Number of previous concussions, Median [IQR] | 1.00 [1.00-2.00] | 2.00 [1.00-2.50] |
| Migraines, n (%) | 2 (5.7) | 2 (20.0) |
| Learning disabilities, n (%) | 3 (8.6) | 1 (10.0) |
| ADHD, n (%) | 6 (1.7) | 1 (10.0) |
| Other developmental disorders, n (%) | 1 (2.9) ^b^ | 0 (0.0) |
| Anxiety, n (%) | 4 (11.4) | 1 (10.0) |
| Depression, n (%) | 3 (8.6) | 1 (10.0) |
| Sleep disorders, n (%) | 1 (2.9) | 0 (0.0) |
| Other mental health diagnoses, n (%) | 1 (2.9) ^c^ | 0 (0.0) |
| **Clinical variables** |  |  |
| Recruitment site, n (%) |  |  |
| ED | 21 (60.0) | 7 (70.0) |
| 360cc | 14 (40.0) | 3 (30.0) |
| Hours post-injury at enrollment, Median [IQR] | 25.95 [3.20-90.50] | 66.19 [12.57-98.45] |
| 5P risk score ^d^, Median [IQR] | 7.00 [6.00-8.00] | 8.00 [7.25-9.00] |
| Low risk, n (%) | 0 (0.0) | 0 (0.0) |
| Medium risk, n (%) | 29 (82.9) | 6 (60.0) |
| High risk, n (%) | 6 (17.1) | 4 (40.0) |
| Loss of consciousness, n (%) | 7 (20.0) | 1 (10.0) |
| Amnesia, n (%) | 11 (36.4) ^mv=1^ | 3 (30.0) |
| Previous experience with mind training therapy, n (%) | 2 (5.7) | 0 (0.0) |
| Prior experience with mindfulness, n (%) | 4 (11.4) | 1 (10.0) |
| Mechanism of injury, n (%) |  |  |
| Fall down stairs | 1 (2.9) | 0 (0.0) |
| Sports | 25 (71.4) | 8 (80.0) |
| Fall from standing, walking, or running | 2 (5.7) | 1 (10.0) |
| Ran into stationary object | 0 (0.0) | 0 (0.0) |
| Other mechanism | 6 (17.1) | 1 (10.0) |
| Missing | 1 | 0 |
| Retrospective HBI, Median [IQR] | 10.50 [4.00-17.00] | 13.00 [6.25-17.50] |
| **Baseline measures, Median [IQR]** |  |  |
| HBI (symptom burden) | 25.00 [16.50-32.00] | 31.50 [23.00-40.00] |
| PedsQL (quality of life) | 81.56 [74.02-90.00] | 75.86 [71.21-88.56] |
| Physical | 90.63 [76.56-99.22] | 89.06 [79.69-93.75] |
| Emotional | 82.50 [65.00-95.00] | 70.00 [57.50-95.00] |
| Social | 95.00 [75.00-100.00] | 95.00 [71.25-100.00] |
| School | 70.00 [60.00-88.75] | 72.50 [56.25-75.00] |
| PedsQL-MFS (fatigue) | 72.92 [61.46-80.56] | 66.67 [49.31-75.35] |
| Cognitive | 75.00 [58.33-90.63] | 64.58 [43.75-80.21] |
| Sleep/Rest | 64.58 [55.21-78.13] | 52.08 [50.00-71.88] |
| General | 81.25 [66.67-87.50] | 79.17 [51.04-92.71] |
| CES-DC (depression) | 4.00 [3.00-8.00] | 4.50 [3.00-8.75] |
| GAD-7 (anxiety) | 4.00 [0.00-8.00] | 3.00 [1.00-9.25] |
| CD-RISC-10 (resilience) | 28.50 [25.00-32.75] | 24.50 [20.00-27.75] |
| CAMM (mindfulness) | 31.50 [26.25-36.50] | 34.50 [32.00-37.00] |
| SEQ-C (self-efficacy) | 85.50 [74.25-95.00] | 81.00 [70.50-92.00] |
| Emotional | 28.00 [23.25-32.50] | 27.50 [22.50-31.50] |
| Social | 30.00 [26.00-33.75] | 27.00 [26.00-31.75] |
| Academic | 28.50 [22.25-32.75] | 26.00 [23.0-30.25] |
| **Outcomes** |  |  |
| Adherence rates over the first 4 weeks, n (%) | 20 (57.1%) | 8 (80.0%) |
| App engagement over the fist 4 weeks (mins), Median [IQR] | 199.17 [64.92-293.84] | 246.32 [172.74-285.03] |

*Notes*. ^a^ MBI-DTx group continued with additional MBI intervention modules and activities for weeks 5-8,^b^ Word retrieval/language delay, ^c^ Unspecified, ^d^ Low risk=0-3, Medium risk=4-8, Higher risk=9-12.

*Abbreviations*: 360cc=360 Concussion Care clinic; CAMM=Child and Adolescent Mindfulness Measure; CES-DC= Center for Epidemiologic Studies Depression Scale for Children; CD-RISC-10=Connor-Davidson Resilience Scale-10; DTx-MBI=Digital Therapeutics Mindfulness-Based Intervention (experimental intervention); ED=Emergency Department; IQR=Interquartile Range [Q1, Q3]; MFS=Multidimensional Fatigue Scale PedsQL; GAD-7=Generalized Anxiety Disorder 7-item scale; HBI=Health Behaviour Inventory; PedsQL=Pediatric Quality of Life Inventory, version 4.0; SEQ-C=Self-Efficacy Questionnaire for Children.

**Table S2. Baseline characteristics, adherence, and attrition rates among Sham participants by study completion status (i.e., those who stopped study participants at the week 4 endpoint versus those who decided to crossover to the MBI condition and continue for four weeks)**

| **Variables** | **4-week study completion**  **N=32** | **Crossover to DTx-MBI ^a^**  **N=11** |
| --- | --- | --- |
| **Demographics** |  |  |
| Age, Median [IQR] | 13.89 [13.13-16.19] | 16.63 [15.34-16.93] |
| Female sex, n (%) | 17 (53.1) | 8 (72.7) |
| **Diagnostic history** |  |  |
| Previous concussion, n (%) | 13 (40.6) | 7 (63.6) |
| Number of previous concussions, Median [IQR] | 1.00 [1.00-2.00] | 2.00 [1.50-3.00] |
| Migraines, n (%) | 2 (6.3) | 0 (0.0) |
| Learning disabilities, n (%) | 5 (15.6) | 1 (9.1) |
| ADHD, n (%) | 2 (6.3) | 2 (18.2) |
| Other developmental disorders, n (%) | 1 (3.1) ^b^ | 0 (0.0) |
| Anxiety, n (%) | 5 (15.6) | 3 (27.3) |
| Depression, n (%) | 0 (0.0) | 1 (9.1) |
| Sleep disorders, n (%) | 0 (0.0) | 0 (0.0) |
| Other mental health diagnoses, n (%) | 0 (0.0) | 0 (0.0) |
| **Clinical variables** |  |  |
| Recruitment site, n (%) |  |  |
| ED | 22 (68.8) | 6 (54.5) |
| 360cc | 10 (31.3) | 5 (45.5) |
| Hours post-injury at enrollment, Median [IQR] | 27.00 [10.45-99.81] | 70.00 [7.26-135.21] |
| 5P risk score ^c^, Median [IQR] | 6.00 [6.00-8.00] | 8.00 [6.00-8.50] |
| Low risk, n (%) | 0 (0.0) | 1 (9.1) |
| Medium risk, n (%) | 25 (78.1) | 7 (63.6) |
| High risk, n (%) | 7 (21.9) | 6 (54.5) |
| Loss of consciousness, n (%) | 4 (12.5) | 1 (9.1) |
| Amnesia, n (%) | 8 (25.0) ^mv=1^ | 6 (54.5) |
| Previous experience with mind training therapy, n (%) | 2 (6.3) | 0 (0.0) |
| Prior experience with mindfulness, n (%) | 5 (15.6) | 1 (9.1) |
| Mechanism of injury, n (%) |  |  |
| Fall down stairs | 1 (3.1) | 0 (0.0) |
| Sports | 21 (65.6) | 8 (72.7) |
| Fall from standing, walking, or running | 4 (12.5) | 1 (9.1) |
| Ran into stationary object | 1 (3.1) | 1 (9.1) |
| Other mechanism | 5 (15.6) | 1 (9.1) |
| Missing | 0 | 0 |
| Retrospective HBI, Median [IQR] | 10.50 [3.75-16.25] | 12.00 [6.00-16.50] |
| **Baseline measures, Median [IQR]** |  |  |
| HBI (symptom burden) | 23.00 [12.75-32.00] | 25.00 [18.00-30.00] |
| PedsQL (quality of life) | 77.19 [67.81-86.33] ^mv=1^ | 85.63 [77.58-90.86] |
| Physical | 87.50 [76.56-93.75] ^mv=1^ | 87.50 [82.91-98.44] |
| Emotional | 70.00 [57.50-85.00] ^mv=1^ | 85.00 [67.50-97.50] |
| Social | 90.00 [70.00-100.00] ^mv=1^ | 100.00 [90.00-100.00] |
| School | 70.00 [55.00-82.50] ^mv=1^ | 75.00 [65.00-90.00] |
| PedsQL-MFS (fatigue) | 69.44 [62.50-80.56] ^mv=1^ | 69.44 [64.58-81.94] |
| Cognitive | 79.17 [58.33-87.50] ^mv=1^ | 75.00 [60.42-91.68] |
| Sleep/Rest | 62.50 [50.00-70.83] ^mv=1^ | 62.50 [52.08-75.00] |
| General | 79.17 [70.83-91.67] ^mv=1^ | 79.17 [66.67-87.50] |
| CES-DC (depression) | 5.00 [3.00-7.25] | 4.00 [4.00, 6.50] |
| GAD-7 (anxiety) | 5.00 [1.00-11.00] ^mv=1^ | 4.00 [2.50-8.00] |
| CD-RISC-10 (resilience) | 28.00 [24.75-31.00] | 29.00 [25.50-31.00] |
| CAMM (mindfulness) | 31.00 [23.50-36.00] ^mv=1^ | 31.00 [26.50-33.50] |
| SEQ-C (self-efficacy) | 82.00 [74.25-90.50] | 87.00 [81.00-89.50] |
| Emotional | 27.00 [24.00-30.00] | 28.00 [25.50-31.00] |
| Social | 28.00 [26.00-32.25] | 30.00 [29.0031.00] |
| Academic | 26.50 [24.00-30.25] | 27.00 [24.50-31.00] |
| **Outcomes** |  |  |
| Adherence rates, n (%) | 19 (59.4) | 5 (45.5) |
| App engagement over the 4 weeks (mins), Median [IQR] | 195.87 [42.37-366.26] | 158.45 [117.88-244.49] |

*Notes*. ^a^ Sham group crossed over to MBI intervention, starting from module 1 for weeks 5-8,^b^ Autism spectrum disorder, ^c^ Low risk=0-3, Medium risk=4-8, Higher risk=9-12, mv=missing values.*Abbreviations*: 360cc=360 Concussion Care clinic; CAMM=Child and Adolescent Mindfulness Measure; CES-DC= Center for Epidemiologic Studies Depression Scale for Children; CD-RISC-10=Connor-Davidson Resilience Scale-10; DTx-MBI=Digital therapeutics Mindfulness-Based Intervention (experimental intervention); ED=Emergency Department; IQR=Interquartile Range [Q1, Q3]; MFS=Multidimensional Fatigue Scale PedsQL; GAD-7=Generalized Anxiety Disorder 7-item scale; HBI=Health Behaviour Inventory; PedsQL=Pediatric Quality of Life Inventory, version 4.0; SEQ-C=Self-Efficacy Questionnaire for Children.

**Table S3. Efficacy signal for outcomes at week 8 post-enrollment**

| **Outcomes, Median [IQR]** | **8-week endpoint (secondary)** | |
| --- | --- | --- |
|  | **DTx-MBI ^a^ (n=9)** | **Sham started MBI at 4 weeks ^b^ (n=10)** |
| HBI (symptom burden) | 4.00 [1.00-21.00] | 13.00 [7.50-19.00] |
| PSAC,^c^ n (%) | 0 (0.0) | 0 (0.0) |
| PedsQL (quality of life) | 95.00 [85.00-98.75] | 87.58 [80.04- 94.69] |
| Physical | 100.00 [90.63-100.00] | 95.31 [81.25-100.00] |
| Emotional | 95.00 [80.00-100.00] | 90.00 [61.25-93.75] |
| Social | 100.00 [50.00-100.00] | 97.50 [91.25-100.00] |
| School | 95.00 [75.00-100.00] | 82.50 [76.25-85.00] |
| PedsQL-MFS (fatigue) | 90.28 [75.00-93.06] | 73.61 [61.46-88.19] |
| Cognitive | 91.67 [75.00- 100.00] | 75.00 [63.54-92.71] |
| Sleep/Rest | 79.17 [66.67-83.33] | 66.67 [59.38-77.08] |
| General | 95.83 [75.00-100.00] | 81.25 [60.42-90.63] |
| CES-DC (depression) | 7.00 [4.00-8.75] | 6.00 [4.25- 9.50] |
| GAD-7 (anxiety) | 1.00 [0.00-3.00] | 2.50 [0.00-5.75] |
| CD-RISC-10 (resilience) | 28.00 [27.00-34.00] | 29.50 [28.25-33.50] |
| CAMM (mindfulness) | 36.00 [27.00-34.00] | 32.50 [21.50-39.75] |
| SEQ-C (self-efficacy) | 89.00 [73.00-100.00] | 89.50 [76.75- 100.75] |
| Emotional | 27.00 [23.00-36.00] | 32.50 [22.75-35.25] |
| Social | 31.00 [25.00-31.00] | 31.50 [25.25-32.75] |
| Academic | 30.00 [27.00-33.00] | 31.00 [25.25-35.00] |
| NIH Toolbox (cognition) |  |  |
| Fluid Cognition composite score (age-corrected) | − | − |
| Flanker (inhibition control, attention) | − | − |
| List Sorting (working memory) | − | − |
| Dimensional Change Card Sort (executive functions) | − | − |
| Pattern Comparison (processing speed) | − | − |
| Picture Sequence (memory) | − | − |
| Agreed to continue to 8 weeks, no. (%) | − | − |

^a^ MBI-DTx group continued with additional MBI intervention modules and activities for weeks 5-8. ^b^ Sham group crossed over to MBI intervention, starting from module 1 for weeks 5-8. ^c^ PSAC was determined through reliable change z scores of ≥1.65 [1]. The reliable change score compares the parent’s retrospective rating of preinjury total symptoms with the child’s ratings reported at the 4-week follow-up, based on a formulae derived from regression analyses in children with orthopedic injuries. *Abbreviations*: CAMM=Child and Adolescent Mindfulness Measure; CES-DC= Center for Epidemiologic Studies Depression Scale for Children; CD-RISC-10=Connor-Davidson Resilience Scale-10; DTx-MBI=Digital Therapeutics Mindfulness-Based Intervention; IQR=Interquartile Range [Q1, Q3]; GAD-7=Generalized Anxiety Disorder 7-item scale; HBI=Health Behaviour Inventory; MFS=Multidimensional Fatigue Scale; NIH=National Institutes of Health; PedsQL=Pediatric Quality of Life Inventory, version 4.0; PSAC=Persisting Symptoms After Concussion; SEQ-C=Self-Efficacy Questionnaire for Children.

1. **Feasibility Outcomes in the MRI Component**

**Procedures**

A subset from each group was prospectively approached and enrolled for a neuroimaging component from February 2023 to June 2024, involving MRI scans pre-intervention (within 72 hours ± 48 hours of enrollment) and post-intervention (week 4). The neuroimaging component of the study was completed at the Royal Ottawa Hospital’s Brain Imaging Centre on a 3-Tesla Siemens MR-PET system (Siemens Biograph mMR, Siemens, Erlangen, Germany). The visit was approximately 90 minutes, including a 45-minute scan.

**Exclusion criteria**

Additional exclusion criteria for the neuroimaging component were the following: (1) previous neurological or select neurodevelopmental disorders such as intellectual disability/ or autism spectrum disorder (history of attention deficit hyperactivity, learning disability, or Tourette’s syndrome was not exclusionary); (2) previous transient ischemic attacks; (3) sedation medication prior to or during ED visit (eg, propofol, ketamine, nitrous oxide, midazolam, benzodiazepines, fentanyl); (4) unable to attend the in-person 72-hour ± 48 hours and 4-week ± 5 days neuroimaging follow-ups; (5) any contraindications to magnetic resonance imaging (MRI; eg, claustrophobia, pregnancy, braces/spacer, metal implants).

**Sample size calculation**

For the nested neuroimaging component, 24 participants (12 per group) are needed for 80% power at the single-voxel level following Desmond and Glover [2]. This number was doubled to adjust for multiple comparisons. Based on our previous study [1] and accounting for 20% attrition, 60 participants were needed.


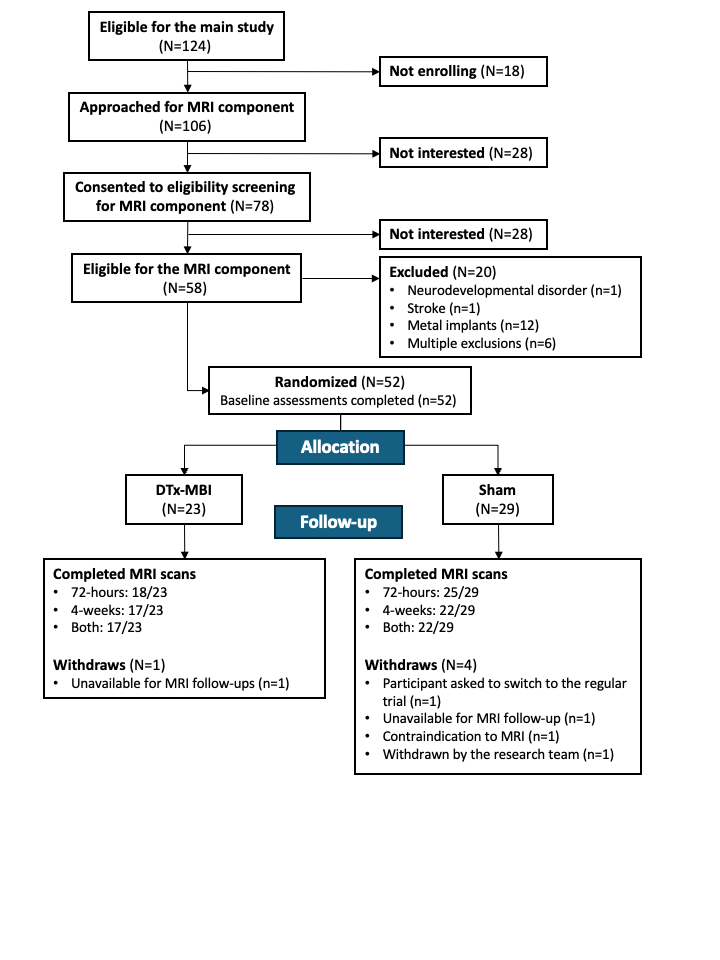


**Figure S1. Participant flow diagram for the neuroimaging component**; DTx-MBI=Digital Therapeutics Mindfulness-Based Intervention; MRI=magnetic resonance imaging.

**Results: Feasibility outcomes for the MRI component**

From the pool of main study participants, 106 were approached for potential enrollment in the optional MRI component. Of these, 73.6% (78/106) expressed interest and underwent screening. Among those screened, 74.4% (58/78; eligibility: green zone) were eligible and provided consent for MRI participation. Of these participants, 89.7% (52/58, recruitment: green zone) were randomized to either DTx-MBI (n=23) or Sham (n=29) conditions. Within the DTx-MBI group, 78.3% (18/23) completed the first MRI scan, 73.9% (17/23) completed the second MRI scan, and 77.9% (17/23) completed both scans (retention: green zone). One participant withdrew from the MRI component due to their inability to attend the first MRI appointment. Within the Sham group, 86.2% (25/29) completed the first MRI scan, 75.9% (22/29) completed the second MRI scan, and 75.9% (22/29, retention: green zone) completed both scans. Four participants withdrew from the MRI component for different reasons (Figure S1).

## References

1. Healey K, Fang Z, Smith A, Zemek R, Ledoux AA. Adolescents with a concussion have altered brain network functional connectivity one month following injury when compared to adolescents with orthopedic injuries. Neuroimage Clin. 2022;36.
2. Desmond JE, Glover GH. Estimating sample size in functional MRI (fMRI) neuroimaging studies: statistical power analyses. J Neurosci Methods. Aug 30, 2002;118(2):115-128.
