# Supplementary material for: Smartphone App–Delivered Mindfulness-Based Intervention for Concussion in Adolescents (MBI-4-mTBI): Feasibility Randomized Controlled Trial
Source: JMIR Rehabil Assist Technol. 2026 May 11;13:e84623. doi: 10.2196/84623 (PMC13160406; doi:10.2196/84623)
Supplement: Checklist 1 [file rehab-v13-e84623-s002.pdf]

CONSORT-EHEALTH (V 1.6.1) - Submission/Publication Form

The CONSORT-EHEALTH checklist is intended for authors of randomized trials evaluating web-based and Internet-based applications/interventions, including mobile interventions, electronic games (incl multiplayer games), social media, certain telehealth applications, and other interactive and/or networked electronic applications. Some of the items (e.g. all subitems under item 5 - description of the intervention) may also be applicable for other study designs.

The goal of the CONSORT EHEALTH checklist and guideline is to be
a) a guide for reporting for authors of RCTs,
b) to form a basis for appraisal of an ehealth trial (in terms of validity)

CONSORT-EHEALTH items/subitems are MANDATORY reporting items for studies published in the Journal of Medical Internet Research and other journals / scientific societies endorsing the checklist.

Items numbered 1., 2., 3., 4a., 4b etc are original CONSORT or CONSORT-NPT (non-pharmacologic treatment) items.
Items with Roman numerals (i., ii, iii, iv etc.) are CONSORT-EHEALTH extensions/clarifications.

As the CONSORT-EHEALTH checklist is still considered in a formative stage, we would ask that you also RATE ON A SCALE OF 1-5 how important/useful you feel each item is FOR THE PURPOSE OF THE CHECKLIST and reporting guideline (optional).

Mandatory reporting items are marked with a red *.
In the textboxes, either copy & paste the relevant sections from your manuscript into this form - please include any quotes from your manuscript in QUOTATION MARKS,
or answer directly by providing additional information not in the manuscript, or elaborating on why the item was not relevant for this study.

YOUR ANSWERS WILL BE PUBLISHED AS A SUPPLEMENTARY FILE TO YOUR PUBLICATION IN JMIR AND ARE CONSIDERED PART OF YOUR PUBLICATION (IF ACCEPTED).
Please fill in these questions diligently. Information will not be copyedited, so please use proper spelling and grammar, use correct capitalization, and avoid abbreviations.

DO NOT FORGET TO SAVE AS PDF _AND_ CLICK THE SUBMIT BUTTON SO YOUR ANSWERS ARE IN OUR DATABASE !!!

Citation Suggestion (if you append the pdf as Appendix we suggest to cite this paper in the caption):
Eysenbach G, CONSORT-EHEALTH Group
CONSORT-EHEALTH: Improving and Standardizing Evaluation Reports of Web-based and Mobile Health Interventions
J Med Internet Res 2011;13(4):e126
URL: http://www.jmir.org/2011/4/e126/
doi: 10.2196/jmir.1923
PMID: 22209829

veronik.sicard@gmail.com Switch account
Draft not saved
Not shared

* Indicates required question

Your name *
First Last

Veronik Sicard

Primary Affiliation (short), City, Country *
University of Toronto, Toronto, Canada

CHEO Research Institute, Ottawa, Canada

Your e-mail address *
abc@gmail.com

vsicard@cheo.on.ca

Title of your manuscript *
Provide the (draft) title of your manuscript.

Smartphone App–Delivered Mindfulness-Based Intervention for Concussion in Adolescents (MBI-4-mTBI): A Feasibility Randomized Controlled Trial

Name of your App/Software/Intervention *
If there is a short and a long/alternate name, write the short name first and add the long name in brackets.

AmDTx

Evaluated Version (if any)
e.g. "V1", "Release 2017-03-01", "Version 2.0.27913"

Non applicable

Language(s) *
What language is the intervention/app in? If multiple languages are available, separate by comma (e.g. "English, French")

English

URL of your Intervention Website or App
e.g. a direct link to the mobile app on app in appstore (itunes, Google Play), or URL of the website. If the intervention is a DVD or hardware, you can also link to an Amazon page.

Your answer

URL of an image/screenshot (optional)

Your answer

Accessibility *
Can an enduser access the intervention presently?

- ( ) access is free and open
- (●) access only for special usergroups, not open
- ( ) access is open to everyone, but requires payment/subscription/in-app purchases
- ( ) app/intervention no longer accessible
- ( ) Other:

Primary Medical Indication/Disease/Condition *
e.g. "Stress", "Diabetes", or define the target group in brackets after the condition, e.g. "Autism (Parents of children with)", "Alzheimers (Informal Caregivers of)"

"Concussion", "Mild traumatic brain injury"

Primary Outcomes measured in trial *
comma-separated list of primary outcomes reported in the trial

Eligibility, Recruitment, Credibility, Retention, A

Secondary/other outcomes
Are there any other outcomes the intervention is expected to affect?

Treatment expectancy, Intervention satisfaction, Safety

Recommended "Dose" *
What do the instructions for users say on how often the app should be used?

- (●) Approximately Daily
- ( ) Approximately Weekly
- ( ) Approximately Monthly
- ( ) Approximately Yearly
- ( ) "as needed"
- ( ) Other:

Approx. Percentage of Users (starters) still using the app as recommended after 3 months *

- (●) unknown / not evaluated
- ( ) 0-10%
- ( ) 11-20%
- ( ) 21-30%
- ( ) 31-40%
- ( ) 41-50%
- ( ) 51-60%
- ( ) 61-70%
- ( ) 71%-80%
- ( ) 81-90%
- ( ) 91-100%
- ( ) Other:

Overall, was the app/intervention effective? *

- ( ) yes: all primary outcomes were significantly better in intervention group vs control
- ( ) partly: SOME primary outcomes were significantly better in intervention group vs control
- ( ) no statistically significant difference between control and intervention
- ( ) potentially harmful: control was significantly better than intervention in one or more outcomes
- ( ) inconclusive: more research is needed
- (●) Other: The study aimed to determine the feasibility of a larger RCT. Not effica

Article Preparation Status/Stage *
At which stage in your article preparation are you currently (at the time you fill in this form)

- ( ) not submitted yet - in early draft status
- ( ) not submitted yet - in late draft status, just before submission
- (●) submitted to a journal but not reviewed yet
- ( ) submitted to a journal and after receiving initial reviewer comments
- ( ) submitted to a journal and accepted, but not published yet
- ( ) published
- ( ) Other:

Journal *
If you already know where you will submit this paper (or if it is already submitted), please provide the journal name (if it is not JMIR, provide the journal name under "other")

- ( ) not submitted yet / unclear where I will submit this
- ( ) Journal of Medical Internet Research (JMIR)
- ( ) JMIR mHealth and UHealth
- ( ) JMIR Serious Games
- ( ) JMIR Mental Health
- ( ) JMIR Public Health
- ( ) JMIR Formative Research
- ( ) Other JMIR sister journal
- (●) Other: JMIR Rehabilitation and Assistive Technologies

Is this a full powered effectiveness trial or a pilot/feasibility trial? *

- (●) Pilot/feasibility
- ( ) Fully powered

Manuscript tracking number *
If this is a JMIR submission, please provide the manuscript tracking number under "other" (The ms tracking number can be found in the submission acknowledgement email, or when you login as author in JMIR. If the paper is already published in JMIR, then the ms tracking number is the four-digit number at the end of the DOI, to be found at the bottom of each published article in JMIR)

- ( ) no ms number (yet) / not (yet) submitted to / published in JMIR
- (●) Other: JRAT ms#84623

TITLE AND ABSTRACT

1a) TITLE: Identification as a randomized trial in the title

1a) Does your paper address CONSORT item 1a? *
I.e does the title contain the phrase "Randomized Controlled Trial"? (if not, explain the reason under "other")

- (●) yes
- ( ) Other:

1a-i) Identify the mode of delivery in the title
Identify the mode of delivery. Preferably use "web-based" and/or "mobile" and/or "electronic game" in the title. Avoid ambiguous terms like "online", "virtual", "interactive". Use "Internet-based" only if Intervention includes non-web-based Internet components (e.g. email), use "computer-based" or "electronic" only if offline products are used. Use "virtual" only in the context of "virtual reality" (3-D worlds). Use "online" only in the context of "online support groups". Complement or substitute product names with broader terms for the class of products (such as "mobile" or "smart phone" instead of "iphone"), especially if the application runs on different platforms.

	1	2	3	4	5	
subitem not at all important	○	○	○	○	●	essential

Clear selection

Does your paper address subitem 1a-i? *
Copy and paste relevant sections from manuscript title (include quotes in quotation marks "like this" to indicate direct quotes from your manuscript), or elaborate on this item by providing additional information not in the ms, or briefly explain why the item is not applicable/relevant for your study

"Smartphone App–Delivered"

1a-ii) Non-web-based components or important co-interventions in title
Mention non-web-based components or important co-interventions in title, if any (e.g., "with telephone support").

	1	2	3	4	5	
subitem not at all important	○	○	○	○	●	essential

Clear selection

Does your paper address subitem 1a-ii?
Copy and paste relevant sections from manuscript title (include quotes in quotation marks "like this" to indicate direct quotes from your manuscript), or elaborate on this item by providing additional information not in the ms, or briefly explain why the item is not applicable/relevant for your study

"Smartphone App–Delivered"

1a-iii) Primary condition or target group in the title
Mention primary condition or target group in the title, if any (e.g., "for children with Type I Diabetes") Example: A Web-based and Mobile Intervention with Telephone Support for Children with Type I Diabetes: Randomized Controlled Trial

	1	2	3	4	5	
subitem not at all important	○	○	○	○	●	essential

Clear selection

Does your paper address subitem 1a-iii? *
Copy and paste relevant sections from manuscript title (include quotes in quotation marks "like this" to indicate direct quotes from your manuscript), or elaborate on this item by providing additional information not in the ms, or briefly explain why the item is not applicable/relevant for your study

Concussion in Adolescents"

1b) ABSTRACT: Structured summary of trial design, methods, results, and conclusions
NPT extension: Description of experimental treatment, comparator, care providers, centers, and blinding status.

1b-i) Key features/functionalities/components of the intervention and comparator in the METHODS section of the ABSTRACT
Mention key features/functionalities/components of the intervention and comparator in the abstract. If possible, also mention theories and principles used for designing the site. Keep in mind the needs of systematic reviewers and indexers by including important synonyms. (Note: Only report in the abstract what the main paper is reporting. If this information is missing from the main body of text, consider adding it)

	1	2	3	4	5	
subitem not at all important	○	○	○	○	●	essential

Clear selection

Does your paper address subitem 1b-i? *
Copy and paste relevant sections from the manuscript abstract (include quotes in quotation marks "like this" to indicate direct quotes from your manuscript), or elaborate on this item by providing additional information not in the ms, or briefly explain why the item is not applicable/relevant for your study

"the experimental group (DTx-MBI) or control group (sham, attention-matched math puzzle game). The DTx-MBI was delivered via AmDTx platform (Mobio Interactive Pte Ltd, Singapore) as a custom-designed 4-to-8-week program of 8 standardized modules for adolescents with concussion, including audio-recorded guided mindfulness exercises, goal setting, journaling, and psychoeducation. The control intervention, delivered through the same interface, excluded mindfulness content and instead featured the open-source game "2048"."

1b-ii) Level of human involvement in the METHODS section of the ABSTRACT
Clarify the level of human involvement in the abstract, e.g., use phrases like "fully automated" vs. "therapist/nurse/care provider/physician-assisted" (mention number and expertise of providers involved, if any). (Note: Only report in the abstract what the main paper is reporting. If this information is missing from the main body of text, consider adding it)

	1	2	3	4	5	
subitem not at all important	●	○	○	○	○	essential

Clear selection

Does your paper address subitem 1b-ii?
Copy and paste relevant sections from the manuscript abstract (include quotes in quotation marks "like this" to indicate direct quotes from your manuscript), or elaborate on this item by providing additional information not in the ms, or briefly explain why the item is not applicable/relevant for your study

Not applicable. It is an app the participants can use.

1b-iii) Open vs. closed, web-based (self-assessment) vs. face-to-face assessments in the METHODS section of the ABSTRACT
Mention how participants were recruited (online vs. offline), e.g., from an open access website or from a clinic or a closed online user group (closed usergroup trial), and clarify if this was a purely web-based trial, or there were face-to-face components (as part of the intervention or for assessment). Clearly say if outcomes were self-assessed through questionnaires (as common in web-based trials). Note: In traditional offline trials, an open trial (open-label trial) is a type of clinical trial in which both the researchers and participants know which treatment is being administered. To avoid confusion, use "blinded" or "unblinded" to indicated the level of blinding instead of "open", as "open" in web-based trials usually refers to "open access" (i.e. participants can self-enrol). (Note: Only report in the abstract what the main paper is reporting. If this information is missing from the main body of text, consider adding it)

	1	2	3	4	5	
subitem not at all important	○	○	○	○	●	essential

Clear selection

Does your paper address subitem 1b-iii?
Copy and paste relevant sections from the manuscript abstract (include quotes in quotation marks "like this" to indicate direct quotes from your manuscript), or elaborate on this item by providing additional information not in the ms, or briefly explain why the item is not applicable/relevant for your study

"This was a Health Canada-regulated, parallel-group, blinded, single-crossover feasibility RCT. Adolescents aged 12 to <18 years presenting to a Pediatric Emergency Department or interdisciplinary concussion clinic within 7 days of a physician-diagnosed concussion were approached for participation from November 2022 to June 2024"

1b-iv) RESULTS section in abstract must contain use data
Report number of participants enrolled/assessed in each group, the use/uptake of the intervention (e.g., attrition/adherence metrics, use over time, number of logins etc.), in addition to primary/secondary outcomes. (Note: Only report in the abstract what the main paper is reporting. If this information is missing from the main body of text, consider adding it)

	1	2	3	4	5	
subitem not at all important	○	○	○	○	●	essential

Clear selection

Does your paper address subitem 1b-iv?
Copy and paste relevant sections from the manuscript abstract (include quotes in quotation marks "like this" to indicate direct quotes from your manuscript), or elaborate on this item by providing additional information not in the ms, or briefly explain why the item is not applicable/relevant for your study

"124/195 (63.6%) screened youth met eligibility criteria. Of these, 99/124 (79.8%) consented and were randomized to either DTx-MBI group (n=49, median[IQR] age=15.28[13.66-16.19] years, 38.8% female) or the Sham group (n=50, median[IQR] age=14.92[13.32-16.71] years, 40.0% female). Credibility was high, with 62/83 (74.7%) of participants scoring above the credibility midpoint (DTx-MBI: 75.0%; Sham: 74.4%). Retention was strong, with 89/99 (89.9%) of participants completing the 4-week outcomes (DTx-MBI: 89.8%; control: 90.0%). Overall adherence was moderate [54/99 (54.5%); DTx-MBI: 59.2%; control: 50.0%], and a little higher among outcome assessment completers [53/89 (59.6%); DTx-MBI: 63.6%; Sham: 55.6%]. Feasibility indicators were similar between groups"

1b-v) CONCLUSIONS/DISCUSSION in abstract for negative trials
Conclusions/Discussions in abstract for negative trials: Discuss the primary outcome - if the trial is negative (primary outcome not changed), and the intervention was not used, discuss whether negative results are attributable to lack of uptake and discuss reasons. (Note: Only report in the abstract what the main paper is reporting. If this information is missing from the main body of text, consider adding it)

	1	2	3	4	5	
subitem not at all important	○	○	○	○	●	essential

Clear selection

Does your paper address subitem 1b-v?
Copy and paste relevant sections from the manuscript abstract (include quotes in quotation marks "like this" to indicate direct quotes from your manuscript), or elaborate on this item by providing additional information not in the ms, or briefly explain why the item is not applicable/relevant for your study

"This feasibility trial supports the implementation of a larger RCT, with modifications to enhance adherence, to rigorously evaluate the clinical efficacy of the DTx-MBI. By targeting modifiable psychological risk factors through a scalable digital platform, DTx-MBI could be a low-burden, cost-effective adjunct to pediatric concussion care."

INTRODUCTION

2a) In INTRODUCTION: Scientific background and explanation of rationale

2a-i) Problem and the type of system/solution
Describe the problem and the type of system/solution that is object of the study: intended as stand-alone intervention vs. incorporated in broader health care program? Intended for a particular patient population? Goals of the intervention, e.g., being more cost-effective to other interventions, replace or complement other solutions? (Note: Details about the intervention are provided in "Methods" under 5)

	1	2	3	4	5	
subitem not at all important	○	○	○	○	●	essential

Clear selection

Does your paper address subitem 2a-i? *
Copy and paste relevant sections from the manuscript (include quotes in quotation marks "like this" to indicate direct quotes from your manuscript), or elaborate on this item by providing additional information not in the ms, or briefly explain why the item is not applicable/relevant for your study

"Early interventions that bolster psychological resilience and emotional regulation may be critical in mitigating symptom burden and preventing chronicity [11,14].", "a recent meta-analysis found that app-based MBIs show small to moderate effectiveness in improving stress, mood, and quality of life among users without a recent concussion [44]. However, the use of short, app-based DTx-MBIs in pediatric concussion remains unexplored"

2a-ii) Scientific background, rationale: What is known about the (type of) system
Scientific background, rationale: What is known about the (type of) system that is the object of the study (be sure to discuss the use of similar systems for other conditions/diagnoses, if appropiate), motivation for the study, i.e. what are the reasons for and what is the context for this specific study, from which stakeholder viewpoint is the study performed, potential impact of findings [2]. Briefly justify the choice of the comparator.

	1	2	3	4	5	
subitem not at all important	○	○	○	○	●	essential

Clear selection

Does your paper address subitem 2a-ii? *
Copy and paste relevant sections from the manuscript (include quotes in quotation marks "like this" to indicate direct quotes from your manuscript), or elaborate on this item by providing additional information not in the ms, or briefly explain why the item is not applicable/relevant for your study

"MBIs have been shown to enhance attention, cognitive flexibility, emotional well-being, and academic performance in children, while reducing anxiety, depressive symptoms, affective reactivity and fear [16]. MBIs reduce default mode network DMN hyperactivity across clinical and pediatric populations,[17–22] and since concussion similarly involves DMN disruptions,[23–27] MBIs may confer neurophysiological benefits post-concussion. MBIs may target several known pre-injury risk factors for PSAC, including mood disorders [28–30], somatization [28,31,32], headache disorders [33], and chronic pain [34]. MBIs may improve fatigue, depression, self-efficacy, and quality of life in individuals of different ages with persistent symptoms following mild-to-moderate traumatic brain injuries, including concussions [35–38]. However, these in-person programs are often costly, time-intensive, and are not always accessible [35–38]. Digital therapeutics (DTx) have emerged as scalable tools for delivering psychological interventions. Evidence supports the effectiveness of brief web-based MBIs in reducing anxiety, perceived stress, and negative mood across different populations [39–43]. Moreover, a recent meta-analysis found that app-based MBIs show small to moderate effectiveness in improving stress, mood, and quality of life among users without a recent concussion [44]."

2b) In INTRODUCTION: Specific objectives or hypotheses

Does your paper address CONSORT subitem 2b? *
Copy and paste relevant sections from the manuscript (include quotes in quotation marks "like this" to indicate direct quotes from your manuscript), or elaborate on this item by providing additional information not in the ms, or briefly explain why the item is not applicable/relevant for your study

"This study aimed to assess the feasibility of conducting a larger randomized controlled trial (RCT) evaluating the effectiveness of DTx-MBI to reduce symptom burden and reduce risk of PSAC in youth with a concussion compared to an attention-matched sham intervention by examining recruitment efficiency, intervention credibility, intervention adherence, and participant retention rates at week 4 post-enrollment. The secondary research objectives were to: (1) Determine the participants' treatment expectations and satisfaction with both interventions; (2) Explore potential efficacy signals across clinical outcomes (ie, concussion symptoms, quality of life, fatigue, anxiety, depression, resiliency, self-efficacy, mindfulness, and cognitive function) to inform future sample size calculations; (3) Compare baseline characteristics, adherence, and attrition rates between: (a) Experimental group: participants who decided to complete the study at 4 weeks and participants who continued for an additional 4 weeks of MBI for weeks 5-8; (b) Control group: participants who decided to complete the study at 4 weeks and who crossed over to the experimental intervention for weeks 5-8; (4) Examine safety by comparing adverse events between interventions; (5) Assess the feasibility of incorporating a neuroimaging component into the larger trial by examining recruitment and retention for the neuroimaging subset, as well as intervention

credibility and intervention adherence among participants who completed neuroimaging."

METHODS

3a) Description of trial design (such as parallel, factorial) including allocation ratio

Does your paper address CONSORT subitem 3a? *

Copy and paste relevant sections from the manuscript (include quotes in quotation marks "like this" to indicate direct quotes from your manuscript), or elaborate on this item by providing additional information not in the ms, or briefly explain why the item is not applicable/relevant for your study

"This Health Canada-regulated feasibility RCT (ClinicalTrials.gov: NCT05105802) used a parallel-group (1:1), blinded, single-crossover design. Participants in the control group who completed the initial 4-week study and wished to pursue the experimental intervention crossed over (modules 1-4) during weeks 5-8, while those in the experimental group could extend their MBI training for an additional 4 weeks (modules 5-8) (Figure 1)."

3b) Important changes to methods after trial commencement (such as eligibility criteria), with reasons

Does your paper address CONSORT subitem 3b? *

Copy and paste relevant sections from the manuscript (include quotes in quotation marks "like this" to indicate direct quotes from your manuscript), or elaborate on this item by providing additional information not in the ms, or briefly explain why the item is not applicable/relevant for your study

Not applicable. We did not make changes to the methods after trial commencement.

3b-i) Bug fixes, Downtimes, Content Changes

Bug fixes, Downtimes, Content Changes: ehealth systems are often dynamic systems. A description of changes to methods therefore also includes important changes made on the intervention or comparator during the trial (e.g., major bug fixes or changes in the functionality or content) (5-iii) and other "unexpected events" that may have influenced study design such as staff changes, system failures/downtimes, etc. [2].

	1	2	3	4	5	
subitem not at all important	○	○	○	○	◉	essential

Clear selection

Does your paper address subitem 3b-i?

Copy and paste relevant sections from the manuscript (include quotes in quotation marks "like this" to indicate direct quotes from your manuscript), or elaborate on this item by providing additional information not in the ms, or briefly explain why the item is not applicable/relevant for your study

The app was not modified over the course of the study.

4a) Eligibility criteria for participants

Does your paper address CONSORT subitem 4a? *

Copy and paste relevant sections from the manuscript (include quotes in quotation marks "like this" to indicate direct quotes from your manuscript), or elaborate on this item by providing additional information not in the ms, or briefly explain why the item is not applicable/relevant for your study

"Adolescents presenting to CHEO's emergency department (ED) or 360cc after sustaining a direct or indirect mild traumatic brain injury (mTBI) were eligible to participate if they: (1) were aged 12 to □18 years; (2) had a diagnosed concussion (by a physician); (3) presented to the ED or 360cc within 7 days of the concussion; (4) had 1 highest level of certainty symptom (eg, loss of consciousness, post-traumatic amnesia) or 2 higher level of certainty symptoms (eg, nausea/vomiting, headache) immediately or within 1 hour of injury from the adapted version of the Centers for Disease Control and Prevention tiered framework [48]; (5) had a score of □4 on the Predicting and Preventing Post-Concussion Problems in Pediatrics (5P) clinical risk score, a validated prediction score to identify those at risk of poorer outcomes at 4 weeks postinjury [3]; (6) were proficient in English.
Patients were excluded if they presented with TBIs with any of the following: (1) Glasgow Coma Scale score of ≤13; (2) trauma-related abnormality on standard neuroimaging studies (if performed) [49]; (3) neurosurgical operative intervention, intubation, or intensive care required; (4) multisystem injuries with the treatment requiring hospital admission, operating room, or procedural sedation in ED; (5) severe neurological developmental delay resulting in communication difficulties; (6) intoxication to alcohol or drugs at the time of ED presentation as per clinician judgment; (7) no clear history of head trauma as the primary event to the concussion (e.g., seizure, migraine attack); (8) prior psychiatric hospitalization; (9) inability to obtain written informed consent or assent; (10) legal guardian not present (certain forms needed to be completed by parents or legal guardians); (11) no internet and mobile or tablet access; and (12) was in psychological therapy at enrollment.
Additional exclusion criteria for the neuroimaging component were the following: (1) previous neurological or select neurodevelopmental disorders such as intellectual disability/ or autism spectrum disorder (history of attention deficit hyperactivity, learning disability, or Tourette's syndrome was not exclusionary); (2) previous transient ischemic attacks; (3) sedation medication prior to or during ED visit (eg, propofol, ketamine, nitrous oxide, midazolam, benzodiazepines, fentanyl); (4) unable to attend the in-person 72-hour ± 48 hours and 4-week ± 5 days neuroimaging follow-ups; (5) any contraindications to magnetic resonance imaging (MRI; eg, claustrophobia, pregnancy, braces/spacer, metal implants)."

4a-i) Computer / Internet literacy

Computer / Internet literacy is often an implicit "de facto" eligibility criterion - this should be explicitly clarified.

	1	2	3	4	5	
subitem not at all important	○	○	○	○	◉	essential

Clear selection

Does your paper address subitem 4a-i?

Copy and paste relevant sections from the manuscript (include quotes in quotation marks "like this" to indicate direct quotes from your manuscript), or elaborate on this item by providing additional information not in the ms, or briefly explain why the item is not applicable/relevant for your study

"(11) no internet and mobile or tablet access"

4a-ii) Open vs. closed, web-based vs. face-to-face assessments:

Open vs. closed, web-based vs. face-to-face assessments: Mention how participants were recruited (online vs. offline), e.g., from an open access website or from a clinic, and clarify if this was a purely web-based trial, or there were face-to-face components (as part of the intervention or for assessment), i.e., to what degree got the study team to know the participant. In online-only trials, clarify if participants were quasi-anonymous and whether having multiple identities was possible or whether technical or logistical measures (e.g., cookies, email confirmation, phone calls) were used to detect/prevent these.

	1	2	3	4	5	
subitem not at all important	○	○	○	○	◉	essential

Clear selection

Does your paper address subitem 4a-ii? *

Copy and paste relevant sections from the manuscript (include quotes in quotation marks "like this" to indicate direct quotes from your manuscript), or elaborate on this item by providing additional information not in the ms, or briefly explain why the item is not applicable/relevant for your study

"The full protocol was published previously [45]. Participants were recruited from the CHEO emergency department (ED) and the 360 Concussion Care (360cc) clinic, an interdisciplinary tertiary health care concussion clinic in Ottawa from October 2022 to June 2024. Briefly, families of adolescents with a physician-diagnosed concussion were approached by a research assistant (RA) to discuss the research study. Interested families completed a screening form on a tablet with the RA, and if eligible and enrolled, filled out electronic questionnaires via Research Electronic Data Capture (REDCap) to collect patient demographics, diagnostic history, injury characteristics, and a concussion symptoms checklist.
The day after the ED or 360cc visit, an RA called participants to randomize them into either the experimental or control group. They provided instructions for installing AmDTx on the participant's mobile device of their choice. Participants completed Day 1 surveys by phone or via REDCap and began the intervention that same day.
At the 4-week mark, participants attended an in-person appointment to complete the cognitive testing at either the CHEO Research Institute or the Royal Ottawa Hospital (for those enrolled in the neuroimaging component; see below). After this visit, participants in the experimental group were offered 4 additional weeks of psychoeducation modules and mindfulness practices. Control group participants were given the option to cross over and begin the DTx-MBI. Follow-up questionnaires were completed on REDCap at 1, 2, 4, and 8 weeks.
A subset from each group was prospectively approached and enrolled for a neuroimaging component from February 2023 to June 2024, involving MRI scans pre-intervention (within 72 hours ± 48 hours of enrollment) and post-intervention (week 4).The neuroimaging component of the study was completed at the Royal Ottawa Hospital's Brain Imaging Centre on a 3-Tesla Siemens MR-PET system (Siemens Biograph mMR, Siemens, Erlangen, Germany). The visit was approximately 90 minutes, including a 45-minute scan."

4a-iii) Information giving during recruitment

Information given during recruitment. Specify how participants were briefed for recruitment and in the informed consent procedures (e.g., publish the informed consent documentation as appendix, see also item X26), as this information may have an effect on user self-selection, user expectation and may also bias results.

	1	2	3	4	5	
subitem not at all important	○	○	○	○	◉	essential

Clear selection

Does your paper address subitem 4a-iii?

Copy and paste relevant sections from the manuscript (include quotes in quotation marks "like this" to indicate direct quotes from your manuscript), or elaborate on this item by providing additional information not in the ms, or briefly explain why the item is not applicable/relevant for your study

"Only the RAs responsible for randomization and app setup were unblinded to participants' group assignments, as this was necessary for intervention setup and delivery. These RAs were blinded to the randomization sequence until the point of group assignment and were not involved in outcome assessment or data analysis.
During the consent process, we gave controlled information to participants about both interventions, while keeping them unaware of the hypothesis, design, and treatment assignment (experimental or control). The study hypothesis and the distinction between experimental and control conditions were disclosed only at the 4-week follow-up, at which point participants could choose to either extend their intervention (DTx-MBI group) or cross over to the experimental condition (Sham group)." We did not publish the informed consent documentation as appendix, but we can.

4b) Settings and locations where the data were collected

Does your paper address CONSORT subitem 4b? *

Copy and paste relevant sections from the manuscript (include quotes in quotation marks "like this" to indicate direct quotes from your manuscript), or elaborate on this item by providing additional information not in the ms, or briefly explain why the item is not applicable/relevant for your study

"Participants were recruited from the CHEO emergency department (ED) and the 360 Concussion Care (360cc) clinic, an interdisciplinary tertiary health care concussion clinic in Ottawa"

4b-i) Report if outcomes were (self-)assessed through online questionnaires

Clearly report if outcomes were (self-)assessed through online questionnaires (as common in web-based trials) or otherwise.

	1	2	3	4	5	
subitem not at all important	○	○	○	○	◉	essential

Clear selection

Does your paper address subitem 4b-i? *

Copy and paste relevant sections from the manuscript (include quotes in quotation marks "like this" to indicate direct quotes from your manuscript), or elaborate on this item by providing additional information not in the ms, or briefly explain why the item is not applicable/relevant for your study

"Follow-up questionnaires were completed on REDCap at 1, 2, 4, and 8 weeks."

4b-ii) Report how institutional affiliations are displayed

Report how institutional affiliations are displayed to potential participants [on ehealth media], as affiliations with prestigious hospitals or universities may affect volunteer rates, use, and reactions with regards to an intervention.(Not a required item – describe only if this may bias results)

	1	2	3	4	5	
subitem not at all important	○	○	○	○	◉	essential

Clear selection

Does your paper address subitem 4b-ii?

Copy and paste relevant sections from the manuscript (include quotes in quotation marks "like this" to indicate direct quotes from your manuscript), or elaborate on this item by providing additional information not in the ms, or briefly explain why the item is not applicable/relevant for your study

It is not in the manuscript. However, the affiliations with CHEO and Research Institute were made clear on the informed consent form.

5) The interventions for each group with sufficient details to allow replication, including how and when they were actually administered

5-i) Mention names, credential, affiliations of the developers, sponsors, and owners

Mention names, credential, affiliations of the developers, sponsors, and owners [6] (if authors/evaluators are owners or developer of the software, this needs to be declared in a "Conflict of interest" section or mentioned elsewhere in the manuscript).

	1	2	3	4	5	
subitem not at all important	○	○	○	○	◉	essential

Clear selection

Does your paper address subitem 5-i?

Copy and paste relevant sections from the manuscript (include quotes in quotation marks "like this" to indicate direct quotes from your manuscript), or elaborate on this item by providing additional information not in the ms, or briefly explain why the item is not applicable/relevant for your study

"The DTx-MBI program was delivered via the AmDTx platform", "The sham digital math puzzle was delivered on the same main interface within AmDTx; "

Conflicts: BJS is the Chief Scientist and CEO of Mobio Interactive PTE LTD (Singapore), the parent company of Mobio Interactive Inc. (Toronto, Canada), and he owned approximately 22% of the company at the time of manuscript submission

5-ii) Describe the history/development process

Describe the history/development process of the application and previous formative evaluations (e.g., focus groups, usability testing), as these will have an impact on adoption/use rates and help with interpreting results.

	1	2	3	4	5	
subitem not at all important	○	○	○	○	◉	essential

Clear selection

Does your paper address subitem 5-ii?

Copy and paste relevant sections from the manuscript (include quotes in quotation marks "like this" to indicate direct quotes from your manuscript), or elaborate on this item by providing additional information not in the ms, or briefly explain why the item is not applicable/relevant for your study

"This reinforces findings from our earlier open-label preliminary study [79,80]. In the mixed-methods evaluation (n=7), credibility, expectancy, and satisfaction ratings were even higher, although this may have been influenced by the small sample size, open-label design, and participants' awareness that they would be asked for feedback during the 4-week interview [79]. "

5-iii) Revisions and updating

Revisions and updating. Clearly mention the date and/or version number of the application/intervention (and comparator, if applicable) evaluated, or describe whether the intervention underwent major changes during the evaluation process, or whether the development and/or content was "frozen" during the trial. Describe dynamic components such as news feeds or changing content which may have an impact on the replicability of the intervention (for unexpected events see item 3b).

	1	2	3	4	5	
subitem not at all important	◉	○	○	○	○	essential

Clear selection

Does your paper address subitem 5-iii?

Copy and paste relevant sections from the manuscript (include quotes in quotation marks "like this" to indicate direct quotes from your manuscript), or elaborate on this item by providing additional information not in the ms, or briefly explain why the item is not applicable/relevant for your study

Not applicable to the study. The journey within the AmDTX app was designed and developed for our use only and no modifications have been made.

5-iv) Quality assurance methods

Provide information on quality assurance methods to ensure accuracy and quality of information provided [1], if applicable.

	1	2	3	4	5	
subitem not at all important	◉	○	○	○	○	essential

Clear selection

Does your paper address subitem 5-iv?

Copy and paste relevant sections from the manuscript (include quotes in quotation marks "like this" to indicate direct quotes from your manuscript), or elaborate on this item by providing additional information not in the ms, or briefly explain why the item is not applicable/relevant for your study

Not applicable to the study.

5-v) Ensure replicability by publishing the source code, and/or providing screenshots/screen-capture video, and/or providing flowcharts of the algorithms used

Ensure replicability by publishing the source code, and/or providing screenshots/screen-capture video, and/or providing flowcharts of the algorithms used. Replicability (i.e., other researchers should in principle be able to replicate the study) is a hallmark of scientific reporting.

	1	2	3	4	5	
subitem not at all important	◉	○	○	○	○	essential

Clear selection

Does your paper address subitem 5-v?

Copy and paste relevant sections from the manuscript (include quotes in quotation marks "like this" to indicate direct quotes from your manuscript), or elaborate on this item by providing additional information not in the ms, or briefly explain why the item is not applicable/relevant for your study

The source code is not published.

5-vi) Digital preservation

Digital preservation: Provide the URL of the application, but as the intervention is likely to change or disappear over the course of the years; also make sure the intervention is archived (Internet Archive, webcitation.org, and/or publishing the source code or screenshots/videos alongside the article). As pages behind login screens cannot be archived, consider creating demo pages which are accessible without login.

	1	2	3	4	5	
subitem not at all important	◉	○	○	○	○	essential

Clear selection

Does your paper address subitem 5-vi?

Copy and paste relevant sections from the manuscript (include quotes in quotation marks "like this" to indicate direct quotes from your manuscript), or elaborate on this item by providing additional information not in the ms, or briefly explain why the item is not applicable/relevant for your study

Not applicable to the study. The journey within the AmDTX app was designed and developed for our use only. Therefore we have the copies.

5-vii) Access

Access: Describe how participants accessed the application, in what setting/context, if they had to pay (or were paid) or not, whether they had to be a member of specific group. If known, describe how participants obtained "access to the platform and Internet" [1]. To ensure access for editors/reviewers/readers, consider to provide a "backdoor" login account or demo mode for reviewers/readers to explore the application (also important for archiving purposes, see vi).

	1	2	3	4	5	
subitem not at all important	○	○	○	○	◉	essential

Clear selection

Does your paper address subitem 5-vii? *

Copy and paste relevant sections from the manuscript (include quotes in quotation marks "like this" to indicate direct quotes from your manuscript), or elaborate on this item by providing additional information not in the ms, or briefly explain why the item is not applicable/relevant for your study

"The day after the ED or 360cc visit, an RA called participants to randomize them into either the experimental or control group. They provided instructions for installing AmDTx on the participant's mobile device of their choice. Participants completed Day 1 surveys by phone or via REDCap and began the intervention that same day. "

5-viii) Mode of delivery, features/functionalities/components of the intervention and comparator, and the theoretical framework

Describe mode of delivery, features/functionalities/components of the intervention and comparator, and the theoretical framework [6] used to design them (instructional strategy [1], behaviour change techniques, persuasive features, etc., see e.g., [7, 8] for terminology). This includes an in-depth description of the content (including where it is coming from and who developed it) [1]," whether [and how] it is tailored to individual circumstances and allows users to track their progress and receive feedback" [6]. This also includes a description of communication delivery channels and – if computer-mediated communication is a component – whether communication was synchronous or asynchronous [6]. It also includes information on presentation strategies [1], including page design principles, average amount of text on pages, presence of hyperlinks to other resources, etc. [1].

	1	2	3	4	5	
subitem not at all important	○	○	○	○	◉	essential

Clear selection

Does your paper address subitem 5-viii? *

Copy and paste relevant sections from the manuscript (include quotes in quotation marks "like this" to indicate direct quotes from your manuscript), or elaborate on this item by providing additional information not in the ms, or briefly explain why the item is not applicable/relevant for your study

"Experimental Group: DTx-MBI
The DTx-MBI program was delivered via the AmDTx platform and consisted of a 4-to-8-week, custom-made program (containing 8 psychoeducation modules) for adolescents with concussions, including goal setting, check-ins on mood and stress, audio-recorded psychoeducation, guided meditations, and journaling. Each standardized module was unlocked as the participant progressed through the program. Participants were encouraged to participate in the DTx-MBI activities for at least 10 minutes every day, for a minimum of 4 days a week over the study period. Those who chose to end their participation at the end of week 4 were immediately directed to module 8 "Reviewing the program and how to maintain practice", while those who decided to continue moved on to modules 5 through 8.
Control Group: Attention-Matched Sham Comparator
The sham digital math puzzle was delivered on the same main interface within AmDTx; however, these participants did not have access to any of the MBI training or psychoeducational content. The sham DTx was the open-source math game called "2048", developed by Gabriele Cirulli [50]. The game required participants to slide numbered tiles on a grid and combine matching numbers, aiming to reach 2048 by creating larger numbers through merges. Each move adds a new tile, and the game ends when the grid is full of no possible merges. The game is programmed to only be accessible for 10 minutes per session.
Snapshot
AmDTx includes four simple tools for tracking patient well-being over time, collectively called the Snapshot, which participants in both groups were prompted to complete before and after each session:(1) 30-second "selfie" video analyzed via photoplethysmography [51] and a deep neural network to quantify psychological stress from heart rate [52] (2) a digital emotion mapping board (circumplex) to capture emotional stress levels; (3) a subjective stress slider, benchmarked against standard psychological surveys [42]; and (4) an open-text personal notes field. The snapshot feature qualified as meaningful engagement, so time spent on it was included in the minimal usage requirement; however, completing the snapshot alone did not fulfill the requirement.
"

5-ix) Describe use parameters

Describe use parameters (e.g., intended "doses" and optimal timing for use). Clarify what instructions or recommendations were given to the user, e.g., regarding timing, frequency, heaviness of use, if any, or was the intervention used ad libitum.

	1	2	3	4	5	
subitem not at all important	○	○	○	○	◉	essential

Clear selection

Does your paper address subitem 5-ix?

Copy and paste relevant sections from the manuscript (include quotes in quotation marks "like this" to indicate direct quotes from your manuscript), or elaborate on this item by providing additional information not in the ms, or briefly explain why the item is not applicable/relevant for your study

"Participants were encouraged to participate in the DTx-MBI activities for at least 10 minutes every day, for a minimum of 4 days a week over the study period. "

5-x) Clarify the level of human involvement

Clarify the level of human involvement (care providers or health professionals, also technical assistance) in the e-intervention or as co-intervention (detail number and expertise of professionals involved, if any, as well as "type of assistance offered, the timing and frequency of the support, how it is initiated, and the medium by which the assistance is delivered". It may be necessary to distinguish between the level of human involvement required for the trial, and the level of human involvement required for a routine application outside of a RCT setting (discuss under item 21 – generalizability).

	1	2	3	4	5	
subitem not at all important	○	○	○	○	◉	essential

Clear selection

Does your paper address subitem 5-x?

Copy and paste relevant sections from the manuscript (include quotes in quotation marks "like this" to indicate direct quotes from your manuscript), or elaborate on this item by providing additional information not in the ms, or briefly explain why the item is not applicable/relevant for your study

It is an app-based intervention. "The full protocol was published previously [45]. "

5-xi) Report any prompts/reminders used

Report any prompts/reminders used: Clarify if there were prompts (letters, emails, phone calls, SMS) to use the application, what triggered them, frequency etc. It may be necessary to distinguish between the level of prompts/reminders required for the trial, and the level of prompts/reminders for a routine application outside of a RCT setting (discuss under item 21 – generalizability).

	1	2	3	4	5	
subitem not at all important	○	○	○	○	◉	essential

Clear selection

Does your paper address subitem 5-xi? *

Copy and paste relevant sections from the manuscript (include quotes in quotation marks "like this" to indicate direct quotes from your manuscript), or elaborate on this item by providing additional information not in the ms, or briefly explain why the item is not applicable/relevant for your study

"The full protocol was published previously [45]. "

5-xii) Describe any co-interventions (incl. training/support)

Describe any co-interventions (incl. training/support): Clearly state any interventions that are provided in addition to the targeted eHealth intervention, as ehealth intervention may not be designed as stand-alone intervention. This includes training sessions and support [1]. It may be necessary to distinguish between the level of training required for the trial, and the level of training for a routine application outside of a RCT setting (discuss under item 21 – generalizability.

	1	2	3	4	5	
subitem not at all important	◉	○	○	○	○	essential

Clear selection

Does your paper address subitem 5-xii? *

Copy and paste relevant sections from the manuscript (include quotes in quotation marks "like this" to indicate direct quotes from your manuscript), or elaborate on this item by providing additional information not in the ms, or briefly explain why the item is not applicable/relevant for your study

Not applicable to the current study.

6a) Completely defined pre-specified primary and secondary outcome measures, including how and when they were assessed

Does your paper address CONSORT subitem 6a? *

Copy and paste relevant sections from the manuscript (include quotes in quotation marks "like this" to indicate direct quotes from your manuscript), or elaborate on this item by providing additional information not in the ms, or briefly explain why the item is not applicable/relevant for your study

"Primary Outcomes
The prespecified feasibility targets for progressing to a definitive trial were informed by established benchmarks from previous feasibility trials [54,55] and our experience recruiting pediatric concussion patients from the ED [56]. We employed a traffic light system to assess the feasibility of proceeding to a full-scale trial [57]. Progression is classified as feasible without modifications (green), feasible with adjustments (amber), or infeasible due to unresolvable issues (red). Advancement to a full-scale trial is determined by the criterion with the least favorable performance. The five prespecified targets (i.e, eligibility, recruitment, credibility, retention, and adherence, along with their corresponding benchmarks) are outlined in Table 1. Credibility was assessed using the Credibility and Expectancy Questionnaire (questions 1-3) at week 1 post-enrollment [58], and retention was defined as completing the Health Behaviour Inventory at the 4-week follow-up [59].
"

Details in Table 1

"Secondary Outcomes
Treatment expectancy was assessed with the Credibility and Expectancy Questionnaire (CEQ, questions 4 to 6) at week 1 after enrollment.[58] Expectancy was considered good if ≥70% participants scored above the midpoint.
Intervention satisfaction was assessed with the modified version (to align with the digital nature of the intervention) of the Client Satisfaction Questionnaire (CSQ) at 4 weeks [60]. The intervention was deemed satisfactory if ≥70% of participants scored above the midpoint on the questionnaire.
Safety of the MBI was determined by capturing and monitoring the worsening of symptoms and adverse events associated with the app or intervention. Adverse events were defined as any unscheduled visits to the ED or primary medical providers because of exacerbation of symptoms over the duration over the study participation. Events were considered intervention-related if they involved symptom provocation during or within 30 minutes of app. use. The determination of whether an adverse event was related to the intervention was made by the Qualified Investigator, based on predefined criteria established by the Data and Safety Monitoring Board and reviewed by the institutional quality assurance and regulatory compliance personnel. Participants were asked about possible adverse events at the 1-, 2-, 4-, and 8-week follow-ups.
Other outcome measures were collected at baseline (in the ED for CHEO participants or on day 1 for 360cc participants) and at 4- and 8-week follow-ups, unless otherwise specified:
- Symptom burden: Health Behavior Inventory (HBI), assessed retrospectively for preinjury and acute postinjury symptoms, with additional assessment at 1 and 2 weeks follow-ups [59];
- Quality of life: Pediatric Quality of Life Inventory (PedsQL™,version 4.0) [61,62];
- Fatigue: PedsQL Multidimensional Fatigue Scale (PedsQL-MFS™, version 4.0) [63,64];
- Depression symptoms: Center for Epidemiologic Studies Depression Scale for Children (CES-DC) [65];
- Anxiety: Generalized Anxiety Disorder 7-item scale (GAD-7) [66];
- Resilience: Connor-Davidson Resilience Scale-10 (CD-RISC-10) [67];
- Mindfulness: Child and Adolescent Mindfulness Measure (CAMM) [68,69];
- Self-efficacy: Self-Efficacy Questionnaire for Children (SEQ-C) [70];
- Cognition: National Institutes of Health Toolbox (NIH Toolbox) [71,72] assessed fluid cognition on week 4 only: Fluid Cognition composite score, Picture Sequence Memory test, List Sorting Working Memory test, Flanker Inhibitory Control and Attention test, Dimensional Change Card Sorting test, and Pattern Comparison Processing Speed test [73].

Adherence
App adherence was quantified based on meaningful in-app activity time, defined as active engagement with core features assigned to each group. For the DTX-MBI group, this included guided meditation exercises, psychoeducational content, and snapshots. For the Sham group, the core features were the "2048" game and snapshots, which served as the control activities for engagement tracking. Any single app session lasting <3 minutes was excluded from adherence calculations, as this duration was deemed insufficient to complete meaningful activities. Weekly adherence was calculated by summing the total seconds of the qualifying app session within each 7-day period over the 4-week study period.
"

6a-i) Online questionnaires: describe if they were validated for online use and apply CHERRIES items to describe how the questionnaires were designed/deployed

If outcomes were obtained through online questionnaires, describe if they were validated for online use and apply CHERRIES items to describe how the questionnaires were designed/deployed [9].

	1	2	3	4	5	
subitem not at all important	◉	○	○	○	○	essential

Clear selection

Does your paper address subitem 6a-i?

Copy and paste relevant sections from manuscript text

All questionnaires were validated. Follow-up questionnaires were completed on REDCap at 1, 2, 4, and 8 weeks.

6a-ii) Describe whether and how "use" (including intensity of use/dosage) was defined/measured/monitored

Describe whether and how "use" (including intensity of use/dosage) was defined/measured/monitored (logins, logfile analysis, etc.). Use/adoption metrics are important process outcomes that should be reported in any ehealth trial.

	1	2	3	4	5	
subitem not at all important	○	○	○	○	◉	essential

Clear selection

Does your paper address subitem 6a-ii?

Copy and paste relevant sections from manuscript text

"App adherence was quantified based on meaningful in-app activity time, defined as active engagement with core features assigned to each group. For the DTX-MBI group, this included guided meditation exercises, psychoeducational content, and snapshots. For the Sham group, the core features were the "2048" game and snapshots, which served as the control activities for engagement tracking. Any single app session lasting <3 minutes was excluded from adherence calculations, as this duration was deemed insufficient to complete meaningful activities. Weekly adherence was calculated by summing the total seconds of the qualifying app session within each 7-day period over the 4-week study period.
"

6a-iii) Describe whether, how, and when qualitative feedback from participants was obtained

Describe whether, how, and when qualitative feedback from participants was obtained (e.g., through emails, feedback forms, interviews, focus groups).

	1	2	3	4	5	
subitem not at all important	◉	○	○	○	○	essential

Clear selection

Does your paper address subitem 6a-iii?

Copy and paste relevant sections from manuscript text

Not applicable to the study

6b) Any changes to trial outcomes after the trial commenced, with reasons

Does your paper address CONSORT subitem 6b? *

Copy and paste relevant sections from the manuscript (include quotes in quotation marks "like this" to indicate direct quotes from your manuscript), or elaborate on this item by providing additional information not in the ms, or briefly explain why the item is not applicable/relevant for your study

Not applicable to the study. No changes were made.

7a) How sample size was determined

NPT: When applicable, details of whether and how the clustering by care provides or centers was addressed

7a-i) Describe whether and how expected attrition was taken into account when calculating the sample size

Describe whether and how expected attrition was taken into account when calculating the sample size.

	1	2	3	4	5	
subitem not at all important	○	○	○	○	◉	essential

Clear selection

Does your paper address subitem 7a-i?

Copy and paste relevant sections from manuscript title (include quotes in quotation marks "like this" to indicate direct quotes from your manuscript), or elaborate on this item by providing additional information not in the ms, or briefly explain why the item is not applicable/relevant for your study

"Detailed sample size justifications can be found in the published protocol [45]. Briefly, sample size estimates were based on feasibility trial guidelines using the traffic light system [57,75]. To achieve >90% power for each of the five feasibility criteria, the highest requirement was adherence (n=55). Accounting for a 20% attrition, 70 participants were needed. Assuming 40% eligibility and 50% recruitment rates, ~350 children were needed to be screened.
For the nested neuroimaging component, 24 participants (12 per group) are needed for 80% power at the single-voxel level following Desmond and Glover [76]. This number was doubled to adjust for multiple comparisons. Based on our previous study [77] and accounting for 20% attrition, 60 participants were needed.
"

7b) When applicable, explanation of any interim analyses and stopping guidelines

Does your paper address CONSORT subitem 7b? *

Copy and paste relevant sections from the manuscript (include quotes in quotation marks "like this" to indicate direct quotes from your manuscript), or elaborate on this item by providing additional information not in the ms, or briefly explain why the item is not applicable/relevant for your study

Not applicable to the study

8a) Method used to generate the random allocation sequence

NPT: When applicable, how care providers were allocated to each trial group

Does your paper address CONSORT subitem 8a? *

Copy and paste relevant sections from the manuscript (include quotes in quotation marks "like this" to indicate direct quotes from your manuscript), or elaborate on this item by providing additional information not in the ms, or briefly explain why the item is not applicable/relevant for your study

"Participants were randomly assigned to one of two groups using a 1:1 allocation ratio stratified by sex: (1) experimental group: DTx-MBI program, or (2) control group: sham digital attention-matched puzzle game. The CHEO Clinical Research Unit provided data management services for this study and retained randomization codes. A statistician who was not involved in the study created and maintained the master list."

8b) Type of randomisation; details of any restriction (such as blocking and block size)

Does your paper address CONSORT subitem 8b? *

Copy and paste relevant sections from the manuscript (include quotes in quotation marks "like this" to indicate direct quotes from your manuscript), or elaborate on this item by providing additional information not in the ms, or briefly explain why the item is not applicable/relevant for your study

"Participants were randomly assigned to one of two groups using a 1:1 allocation ratio stratified by sex: (1) experimental group: DTx-MBI program, or (2) control group: sham digital attention-matched puzzle game. The CHEO Clinical Research Unit provided data management services for this study and retained randomization codes. A statistician who was not involved in the study created and maintained the master list. "

9) Mechanism used to implement the random allocation sequence (such as sequentially numbered containers), describing any steps taken to conceal the sequence until interventions were assigned

Does your paper address CONSORT subitem 9? *

Copy and paste relevant sections from the manuscript (include quotes in quotation marks "like this" to indicate direct quotes from your manuscript), or elaborate on this item by providing additional information not in the ms, or briefly explain why the item is not applicable/relevant for your study

"Participants were randomly assigned to one of two groups using a 1:1 allocation ratio stratified by sex: (1) experimental group: DTx-MBI program, or (2) control group: sham digital attention-matched puzzle game. The CHEO Clinical Research Unit provided data management services for this study and retained randomization codes. A statistician who was not involved in the study created and maintained the master list. "

10) Who generated the random allocation sequence, who enrolled participants, and who assigned participants to interventions

Does your paper address CONSORT subitem 10? *

Copy and paste relevant sections from the manuscript (include quotes in quotation marks "like this" to indicate direct quotes from your manuscript), or elaborate on this item by providing additional information not in the ms, or briefly explain why the item is not applicable/relevant for your study

"Participants were randomly assigned to one of two groups using a 1:1 allocation ratio stratified by sex: (1) experimental group: DTx-MBI program, or (2) control group: sham digital attention-matched puzzle game. The CHEO Clinical Research Unit provided data management services for this study and retained randomization codes. A statistician who was not involved in the study created and maintained the master list.
Only the RAs responsible for randomization and app setup were unblinded to participants' group assignments, as this was necessary for intervention setup and delivery. These RAs were blinded to the randomization sequence until the point of group assignment and were not involved in outcome assessment or data analysis.
During the consent process, we gave controlled information to participants about both interventions, while keeping them unaware of the hypothesis, design, and treatment assignment (experimental or control). The study hypothesis and the distinction between experimental and control conditions were disclosed only at the 4-week follow-up, at which point participants could choose to either extend their intervention (DTx-MBI group) or cross over to the experimental condition (Sham group).
"

11a) If done, who was blinded after assignment to interventions (for example, participants, care providers, those assessing outcomes) and how

NPT: Whether or not administering co-interventions were blinded to group assignment

11a-i) Specify who was blinded, and who wasn't

Specify who was blinded, and who wasn't. Usually, in web-based trials it is not possible to blind the participants [1, 3] (this should be clearly acknowledged), but it may be possible to blind outcome assessors, those doing data analysis or those administering co-interventions (if any).

	1	2	3	4	5	
subitem not at all important	○	○	○	○	◉	essential

Clear selection

Does your paper address subitem 11a-i? *

Copy and paste relevant sections from the manuscript (include quotes in quotation marks "like this" to indicate direct quotes from your manuscript), or elaborate on this item by providing additional information not in the ms, or briefly explain why the item is not applicable/relevant for your study

"Participants were randomly assigned to one of two groups using a 1:1 allocation ratio stratified by sex: (1) experimental group: DTx-MBI program, or (2) control group: sham digital attention-matched puzzle game. The CHEO Clinical Research Unit provided data management services for this study and retained randomization codes. A statistician who was not involved in the study created and maintained the master list.
Only the RAs responsible for randomization and app setup were unblinded to participants' group assignments, as this was necessary for intervention setup and delivery. These RAs were blinded to the randomization sequence until the point of group assignment and were not involved in outcome assessment or data analysis.
During the consent process, we gave controlled information to participants about both interventions, while keeping them unaware of the hypothesis, design, and treatment assignment (experimental or control). The study hypothesis and the distinction between experimental and control conditions were disclosed only at the 4-week follow-up, at which point participants could choose to either extend their intervention (DTx-MBI group) or cross over to the experimental condition (Sham group).
"

11a-ii) Discuss e.g., whether participants knew which intervention was the "intervention of interest" and which one was the "comparator"

Informed consent procedures (4a-ii) can create biases and certain expectations - discuss e.g., whether participants knew which intervention was the "intervention of interest" and which one was the "comparator".

	1	2	3	4	5	
subitem not at all important	○	○	○	○	◉	essential

Clear selection

Does your paper address subitem 11a-ii?

Copy and paste relevant sections from the manuscript (include quotes in quotation marks "like this" to indicate direct quotes from your manuscript), or elaborate on this item by providing additional information not in the ms, or briefly explain why the item is not applicable/relevant for your study

"Participants were randomly assigned to one of two groups using a 1:1 allocation ratio stratified by sex: (1) experimental group: DTx-MBI program, or (2) control group: sham digital attention-matched puzzle game. The CHEO Clinical Research Unit provided data management services for this study and retained randomization codes. A statistician who was not involved in the study created and maintained the master list.
Only the RAs responsible for randomization and app setup were unblinded to participants' group assignments, as this was necessary for intervention setup and delivery. These RAs were blinded to the randomization sequence until the point of group assignment and were not involved in outcome assessment or data analysis.
During the consent process, we gave controlled information to participants about both interventions, while keeping them unaware of the hypothesis, design, and treatment assignment (experimental or control). The study hypothesis and the distinction between experimental and control conditions were disclosed only at the 4-week follow-up, at which point participants could choose to either extend their intervention (DTx-MBI group) or cross over to the experimental condition (Sham group)."

11b) If relevant, description of the similarity of interventions

(this item is usually not relevant for ehealth trials as it refers to similarity of a placebo or sham intervention to a active medication/intervention)

Does your paper address CONSORT subitem 11b? *

Copy and paste relevant sections from the manuscript (include quotes in quotation marks "like this" to indicate direct quotes from your manuscript), or elaborate on this item by providing additional information not in the ms, or briefly explain why the item is not applicable/relevant for your study

Not applicable to the study

12a) Statistical methods used to compare groups for primary and secondary outcomes

NPT: When applicable, details of whether and how the clustering by care providers or centers was addressed

Does your paper address CONSORT subitem 12a? *

Copy and paste relevant sections from the manuscript (include quotes in quotation marks "like this" to indicate direct quotes from your manuscript), or elaborate on this item by providing additional information not in the ms, or briefly explain why the item is not applicable/relevant for your study

"Descriptive statistics were used to summarize participant characteristics, with medians reported for continuous variables and frequencies for categorical variables. Feasibility outcomes were reported as proportions. For outcome measures at 4 and 8 weeks, medians and interquartile ranges were calculated for each group.
In a post-hoc exploratory analysis, a multiple regression was conducted to examine factors associated with app engagement (in seconds, square root transformed to address non-normality and improve model assumptions) over the 4-week intervention period. The regression model included group allocation, age, sex, and pre-injury ratings of anxiety, depression, mindfulness, and self-efficacy.
All statistical analyses were conducted on R version 4.4.1. (2024) [74]."

12a-i) Imputation techniques to deal with attrition / missing values

Imputation techniques to deal with attrition / missing values: Not all participants will use the intervention/comparator as intended and attrition is typically high in ehealth trials. Specify how participants who did not use the application or dropped out from the trial were treated in the statistical analysis (a complete case analysis is strongly discouraged, and simple imputation techniques such as LOCF may also be problematic [4]).

	1	2	3	4	5	
subitem not at all important	○	○	○	○	◉	essential

Clear selection

Does your paper address subitem 12a-i? *

Copy and paste relevant sections from the manuscript (include quotes in quotation marks "like this" to indicate direct quotes from your manuscript), or elaborate on this item by providing additional information not in the ms, or briefly explain why the item is not applicable/relevant for your study

Not applicable to the study

12b) Methods for additional analyses, such as subgroup analyses and adjusted analyses

Does your paper address CONSORT subitem 12b? *

Copy and paste relevant sections from the manuscript (include quotes in quotation marks "like this" to indicate direct quotes from your manuscript), or elaborate on this item by providing additional information not in the ms, or briefly explain why the item is not applicable/relevant for your study

Not applicable to the study

X26) REB/IRB Approval and Ethical Considerations [recommended as subheading under "Methods"] (not a CONSORT item)

X26-i) Comment on ethics committee approval

	1	2	3	4	5	
subitem not at all important	○	○	○	○	◉	essential

Clear selection

Does your paper address subitem X26-i?

Copy and paste relevant sections from the manuscript (include quotes in quotation marks "like this" to indicate direct quotes from your manuscript), or elaborate on this item by providing additional information not in the ms, or briefly explain why the item is not applicable/relevant for your study

"This study was approved by the Research Ethics Board (protocol #20/720X) of the Children's Hospital of Eastern Ontario (CHEO) Research Institute in August 2020. Authorization from Health Canada was granted in July 2022. "

x26-ii) Outline informed consent procedures

Outline informed consent procedures e.g., if consent was obtained offline or online (how? Checkbox, etc.?), and what information was provided (see 4a-ii). See [6] for some items to be included in informed consent documents.

	1	2	3	4	5	
subitem not at all important	◉	○	○	○	○	essential

Clear selection

Does your paper address subitem X26-ii?

Copy and paste relevant sections from the manuscript (include quotes in quotation marks "like this" to indicate direct quotes from your manuscript), or elaborate on this item by providing additional information not in the ms, or briefly explain why the item is not applicable/relevant for your study

"The full protocol was published previously [45]. Participants were recruited from the CHEO emergency department (ED) and the 360 Concussion Care (360cc) clinic, an interdisciplinary tertiary health care concussion clinic in Ottawa from October 2022 to June 2024. Briefly, families of adolescents with a physician-diagnosed concussion were approached by a research assistant (RA) to discuss the research study. Interested families completed a screening form on a tablet with the RA, and if eligible and enrolled, filled out electronic questionnaires via Research Electronic Data Capture (REDCap) to collect patient demographics, diagnostic history, injury characteristics, and a concussion symptoms checklist. "

X26-iii) Safety and security procedures

Safety and security procedures, incl. privacy considerations, and any steps taken to reduce the likelihood or detection of harm (e.g., education and training, availability of a hotline)

	1	2	3	4	5	
subitem not at all important	○	◉	○	○	○	essential

Clear selection

Does your paper address subitem X26-iii?

Copy and paste relevant sections from the manuscript (include quotes in quotation marks "like this" to indicate direct quotes from your manuscript), or elaborate on this item by providing additional information not in the ms, or briefly explain why the item is not applicable/relevant for your study

"Safety of the MBI was determined by capturing and monitoring the worsening of symptoms and adverse events associated with the app or intervention. Adverse events were defined as any unscheduled visits to the ED or primary medical providers because of exacerbation of symptoms over the duration over the study participation. Events were considered intervention-related if they involved symptom provocation during or within 30 minutes of app. use. The determination of whether an adverse event was related to the intervention was made by the Qualified Investigator, based on predefined criteria established by the Data and Safety Monitoring Board and reviewed by the institutional quality assurance and regulatory compliance personnel. Participants were asked about possible adverse events at the 1-, 2-, 4-, and 8-week follow-ups. "

RESULTS

13a) For each group, the numbers of participants who were randomly assigned, received intended treatment, and were analysed for the primary outcome

NPT: The number of care providers or centers performing the intervention in each group and the number of patients treated by each care provider in each center

Does your paper address CONSORT subitem 13a? *

Copy and paste relevant sections from the manuscript (include quotes in quotation marks "like this" to indicate direct quotes from your manuscript), or elaborate on this item by providing additional information not in the ms, or briefly explain why the item is not applicable/relevant for your study

"Of 124 eligible adolescents, 99 participants were randomized to DTx-MBI (n=49; median age [IQR]=15.28 [13.66-16.19]; 61.2% female) or the Sham (n=50; median age [IQR]=14.92 [13.32-16.71]; 60.0% female); 6 declined consent and 19 were not randomized for various factors, including inability to establish contact within the allotted timeframe (Figure 2). 65 participants were recruited from the ED and 34 from 360cc. Four participants (DTx-MBI=2; Sham=2) withdrew from the study after randomization, and 6 participants (DTx-MBI=3; Sham=3) were lost to follow-up (Figure 2). Demographics and clinical characteristics were comparable between groups among all randomized participants and those who completed the 4-week follow-up (see Table 2)."

13b) For each group, losses and exclusions after randomisation, together with reasons

Does your paper address CONSORT subitem 13b? (NOTE: Preferably, this is shown in a CONSORT flow diagram) *

Copy and paste relevant sections from the manuscript (include quotes in quotation marks "like this" to indicate direct quotes from your manuscript), or elaborate on this item by providing additional information not in the ms, or briefly explain why the item is not applicable/relevant for your study

"Of 124 eligible adolescents, 99 participants were randomized to DTx-MBI (n=49; median age [IQR]=15.28 [13.66-16.19]; 61.2% female) or the Sham (n=50; median age [IQR]=14.92 [13.32-16.71]; 60.0% female); 6 declined consent and 19 were not randomized for various factors, including inability to establish contact within the allotted timeframe (Figure 2). 65 participants were recruited from the ED and 34 from 360cc. Four participants (DTx-MBI=2; Sham=2) withdrew from the study after randomization, and 6 participants (DTx-MBI=3; Sham=3) were lost to follow-up (Figure 2). Demographics and clinical characteristics were comparable between groups among all randomized participants and those who completed the 4-week follow-up (see Table 2)."

Table 2 is the CONSORT flow diagram

13b-i) Attrition diagram

Strongly recommended: An attrition diagram (e.g., proportion of participants still logging in or using the intervention/comparator in each group plotted over time, similar to a survival curve) or other figures or tables demonstrating usage/dose/engagement.

	1	2	3	4	5	
subitem not at all important	○	○	○	○	◉	essential

Clear selection

Does your paper address subitem 13b-i?

Copy and paste relevant sections from the manuscript or cite the figure number if applicable (include quotes in quotation marks "like this" to indicate direct quotes from your manuscript), or elaborate on this item by providing additional information not in the ms, or briefly explain why the item is not applicable/relevant for your study

"Among participants who completed the intervention, weekly adherence rates (ie, meeting the threshold of ≥10 minutes of "meaningful" app engagement for 4 days over a 7-day week) declined from week 1 (DTx-MBI: 50.0%; Sham: 68.9%) to week 4 (DTx-MBI: 38.6%; Sham: 37.8%). Similarly, median weekly engagement time decreased from 40.9 minutes [IQR: 20.2-89.9] to 17.1 minutes [IQR: 0-76.8] in the DTx-MBI group and from 75.2 minutes [IQR: 26.2-108.1] to 12.6 minutes [0-60.0] in Sham group over the 4 weeks (Figure 3)."

14a) Dates defining the periods of recruitment and follow-up

Does your paper address CONSORT subitem 14a? *

Copy and paste relevant sections from the manuscript (include quotes in quotation marks "like this" to indicate direct quotes from your manuscript), or elaborate on this item by providing additional information not in the ms, or briefly explain why the item is not applicable/relevant for your study

" from October 2022 to June 2024. "

14a-i) Indicate if critical "secular events" fell into the study period

Indicate if critical "secular events" fell into the study period, e.g., significant changes in Internet resources available or "changes in computer hardware or Internet delivery resources"

	1	2	3	4	5	
subitem not at all important	○	○	◉	○	○	essential

Clear selection

Clear selection

Does your paper address subitem 14a-i?

Copy and paste relevant sections from the manuscript (include quotes in quotation marks "like this" to indicate direct quotes from your manuscript), or elaborate on this item by providing additional information not in the ms, or briefly explain why the item is not applicable/relevant for your study

Not applicable to the study as explained above

14b) Why the trial ended or was stopped (early)

Does your paper address CONSORT subitem 14b? *

Copy and paste relevant sections from the manuscript (include quotes in quotation marks "like this" to indicate direct quotes from your manuscript), or elaborate on this item by providing additional information not in the ms, or briefly explain why the item is not applicable/relevant for your study

Not applicable to the study

15) A table showing baseline demographic and clinical characteristics for each group

NPT: When applicable, a description of care providers (case volume, qualification, expertise, etc.) and centers (volume) in each group

Does your paper address CONSORT subitem 15? *

Copy and paste relevant sections from the manuscript (include quotes in quotation marks "like this" to indicate direct quotes from your manuscript), or elaborate on this item by providing additional information not in the ms, or briefly explain why the item is not applicable/relevant for your study

This is presented in Table 2

15-i) Report demographics associated with digital divide issues

In ehealth trials it is particularly important to report demographics associated with digital divide issues, such as age, education, gender, social-economic status, computer/Internet/ehealth literacy of the participants, if known.

subitem not at all important ○ 1 ○ 2 ● 3 ○ 4 ○ 5 essential

Clear selection

Does your paper address subitem 15-i? *

Copy and paste relevant sections from the manuscript (include quotes in quotation marks "like this" to indicate direct quotes from your manuscript), or elaborate on this item by providing additional information not in the ms, or briefly explain why the item is not applicable/relevant for your study

Not applicable to the study.

16) For each group, number of participants (denominator) included in each analysis and whether the analysis was by original assigned groups

16-i) Report multiple "denominators" and provide definitions

Report multiple "denominators" and provide definitions: Report N's (and effect sizes) "across a range of study participation [and use] thresholds" [1], e.g., N exposed, N consented, N used more than x times, N used more than y weeks, N participants "used" the intervention/comparator at specific pre-defined time points of interest (in absolute and relative numbers per group). Always clearly define "use" of the intervention.

subitem not at all important ○ 1 ○ 2 ○ 3 ○ 4 ● 5 essential

Clear selection

Does your paper address subitem 16-i? *

Copy and paste relevant sections from the manuscript (include quotes in quotation marks "like this" to indicate direct quotes from your manuscript), or elaborate on this item by providing additional information not in the ms, or briefly explain why the item is not applicable/relevant for your study

"Of 124 eligible adolescents, 99 participants were randomized to DTx-MBI (n=49; median age [IQR]=15.28 [13.66-16.19]; 61.2% female) or the Sham (n=50; median age [IQR]=14.92 [13.32-16.71]; 60.0% female)."

16-ii) Primary analysis should be intent-to-treat

Primary analysis should be intent-to-treat, secondary analyses could include comparing only "users", with the appropriate caveats that this is no longer a randomized sample (see 18-i).

subitem not at all important ○ 1 ○ 2 ○ 3 ○ 4 ○ 5 essential

Does your paper address subitem 16-ii?

Copy and paste relevant sections from the manuscript (include quotes in quotation marks "like this" to indicate direct quotes from your manuscript), or elaborate on this item by providing additional information not in the ms, or briefly explain why the item is not applicable/relevant for your study

Your answer

17a) For each primary and secondary outcome, results for each group, and the estimated effect size and its precision (such as 95% confidence interval)

Does your paper address CONSORT subitem 17a? *

Copy and paste relevant sections from the manuscript (include quotes in quotation marks "like this" to indicate direct quotes from your manuscript), or elaborate on this item by providing additional information not in the ms, or briefly explain why the item is not applicable/relevant for your study

Your answer

17a-i) Presentation of process outcomes such as metrics of use and intensity of use

In addition to primary/secondary (clinical) outcomes, the presentation of process outcomes such as metrics of use and intensity of use (dose, exposure) and their operational definitions is critical. This does not only refer to metrics of attrition (13-b) (often a binary variable), but also to more continuous exposure metrics such as "average session length". These must be accompanied by a technical description how a metric like a "session" is defined (e.g., timeout after idle time) [1] (report under item 6a).

subitem not at all important ○ 1 ○ 2 ○ 3 ○ 4 ● 5 essential

Clear selection

Does your paper address subitem 17a-i?

Copy and paste relevant sections from the manuscript (include quotes in quotation marks "like this" to indicate direct quotes from your manuscript), or elaborate on this item by providing additional information not in the ms, or briefly explain why the item is not applicable/relevant for your study

Primary Outcomes
The prespecified feasibility targets for progressing to a definitive trial were informed by established benchmarks from previous feasibility trials [54,55] and our experience recruiting pediatric concussion patients from the ED [56]. We employed a traffic light system to assess the feasibility of proceeding to a full-scale trial [57]. Progression is classified as feasible without modifications (green), feasible with adjustments (amber), or infeasible due to unresolvable issues (red). Advancement to a full-scale trial is determined by the criterion with the least favorable performance. The five prespecified targets (i.e, eligibility, recruitment, credibility, retention, and adherence, along with their corresponding benchmarks) are outlined in Table 1. Credibility was assessed using the Credibility and Expectancy Questionnaire (questions 1-3) at week 1 post-enrollment [58], and retention was defined as completing the Health Behaviour Inventory at the 4-week follow-up [59].

Table 1

Secondary Outcomes
Treatment expectancy was assessed with the Credibility and Expectancy Questionnaire (CEQ, questions 4 to 6) at week 1 after enrollment.[58] Expectancy was considered good if ≥70% participants scored above the midpoint.
Intervention satisfaction was assessed with the modified version (to align with the digital nature of the intervention) of the Client Satisfaction Questionnaire (CSQ) at 4 weeks [60]. The intervention was deemed satisfactory if ≥70% of participants scored above the midpoint on the questionnaire.
Safety of the MBI was determined by capturing and monitoring the worsening of symptoms and adverse events associated with the app or intervention. Adverse events were defined as any unscheduled visits to the ED or primary medical providers because of exacerbation of symptoms over the duration over the study participation. Events were considered intervention-related if they involved symptom provocation during or within 30 minutes of app. use. The determination of whether an adverse event was related to the intervention was made by the Qualified Investigator, based on predefined criteria established by the Data and Safety Monitoring Board and reviewed by the institutional quality assurance and regulatory compliance personnel. Participants were asked about possible adverse events at the 1-, 2-, 4-, and 8-week follow-ups.
Other outcome measures were collected at baseline (in the ED for CHEO participants or on day 1 for 360cc participants) and at 4- and 8-week follow-ups, unless otherwise specified:
- Symptom burden: Health Behavior Inventory (HBI), assessed retrospectively for preinjury and acute postinjury symptoms, with additional assessment at 1 and 2 weeks follow-ups [59];
- Quality of life: Pediatric Quality of Life Inventory (PedsQL™,version 4.0) [61,62];
- Fatigue: PedsQL Multidimensional Fatigue Scale (PedsQL-MFS™, version 4.0) [63,64];
- Depression symptoms: Center for Epidemiologic Studies Depression Scale for Children (CES-DC) [65];
- Anxiety: Generalized Anxiety Disorder 7-item scale (GAD-7) [66];
- Resilience: Connor-Davidson Resilience Scale-10 (CD-RISC-10) [67];
- Mindfulness: Child and Adolescent Mindfulness Measure (CAMM) [68,69];
- Self-efficacy: Self-Efficacy Questionnaire for Children (SEQ-C) [70];
- Cognition: National Institutes of Health Toolbox (NIH Toolbox) [71,72] assessed fluid cognition on week 4 only: Fluid Cognition composite score, Picture Sequence Memory test, List Sorting Working Memory test, Flanker Inhibitory Control and Attention test, Dimensional Change Card Sorting test, and Pattern Comparison Processing Speed test [73].
Adherence
App adherence was quantified based on meaningful in-app activity time, defined as active engagement with core features assigned to each group. For the DTX-MBI group, this included guided meditation exercises, psychoeducational content, and snapshots. For the Sham group, the core features were the "2048" game and snapshots, which served as the control activities for engagement tracking. Any single app session lasting <3 minutes was excluded from adherence calculations, as this duration was deemed insufficient to complete meaningful activities. Weekly adherence was calculated by summing the total seconds of the qualifying app session within each 7-day period over the 4-week study period.

17b) For binary outcomes, presentation of both absolute and relative effect sizes is recommended

Does your paper address CONSORT subitem 17b? *

Copy and paste relevant sections from the manuscript (include quotes in quotation marks "like this" to indicate direct quotes from your manuscript), or elaborate on this item by providing additional information not in the ms, or briefly explain why the item is not applicable/relevant for your study

Non applicable

Your answer must have a minimum of 25 characters.

18) Results of any other analyses performed, including subgroup analyses and adjusted analyses, distinguishing pre-specified from exploratory

Does your paper address CONSORT subitem 18? *

Copy and paste relevant sections from the manuscript (include quotes in quotation marks "like this" to indicate direct quotes from your manuscript), or elaborate on this item by providing additional information not in the ms, or briefly explain why the item is not applicable/relevant for your study

In a post-hoc exploratory analysis, a multiple regression was conducted to examine factors associated with app engagement (in seconds, square root transformed to address non-normality and improve model assumptions) over the 4-week intervention period. The regression model included group allocation, age, sex, and pre-injury ratings of anxiety, depression, mindfulness, and self-efficacy.

18-i) Subgroup analysis of comparing only users

A subgroup analysis of comparing only users is not uncommon in ehealth trials, but if done, it must be stressed that this is a self-selected sample and no longer an unbiased sample from a randomized trial (see 16-iii).

subitem not at all important ○ 1 ○ 2 ○ 3 ○ 4 ● 5 essential

Clear selection

Does your paper address subitem 18-i?

Copy and paste relevant sections from the manuscript (include quotes in quotation marks "like this" to indicate direct quotes from your manuscript), or elaborate on this item by providing additional information not in the ms, or briefly explain why the item is not applicable/relevant for your study

Retention was really good, so non applicable

19) All important harms or unintended effects in each group
(for specific guidance see CONSORT for harms)

Does your paper address CONSORT subitem 19? *

Copy and paste relevant sections from the manuscript (include quotes in quotation marks "like this" to indicate direct quotes from your manuscript), or elaborate on this item by providing additional information not in the ms, or briefly explain why the item is not applicable/relevant for your study

Two participants from the DTx-MBI group reported unscheduled medical visits during their study participation, but no visits met our predefined safety criteria for adverse events associated with the use of the application. Specifically, the first participant went to the ED for sustaining a second concussion during week 3. The second participant went to the ED for worsening of concussion symptoms, but had not yet started using the AmDTx app.

19-i) Include privacy breaches, technical problems

Include privacy breaches, technical problems. This does not only include physical "harm" to participants, but also incidents such as perceived or real privacy breaches [1], technical problems, and other unexpected/unintended incidents. "Unintended effects" also includes unintended positive effects [2].

subitem not at all important ○ 1 ○ 2 ○ 3 ○ 4 ● 5 essential

Clear selection

Does your paper address subitem 19-i?

Copy and paste relevant sections from the manuscript (include quotes in quotation marks "like this" to indicate direct quotes from your manuscript), or elaborate on this item by providing additional information not in the ms, or briefly explain why the item is not applicable/relevant for your study

No breaches happened

Your answer must have a minimum of 25 characters.

19-ii) Include qualitative feedback from participants or observations from staff/researchers

Include qualitative feedback from participants or observations from staff/researchers, if available, on strengths and shortcomings of the application, especially if they point to unintended/unexpected effects or uses. This includes (if available) reasons for why people did or did not use the application as intended by the developers.

subitem not at all important ○ 1 ○ 2 ● 3 ○ 4 ○ 5 essential

Clear selection

Does your paper address subitem 19-ii?

Copy and paste relevant sections from the manuscript (include quotes in quotation marks "like this" to indicate direct quotes from your manuscript), or elaborate on this item by providing additional information not in the ms, or briefly explain why the item is not applicable/relevant for your study

This was done in Phase 1 acceptability (published elsewhere)

DISCUSSION

22) Interpretation consistent with results, balancing benefits and harms, and considering other relevant evidence

NPT: In addition, take into account the choice of the comparator, lack of or partial blinding, and unequal expertise of care providers or centers in each group

22-i) Restate study questions and summarize the answers suggested by the data, starting with primary outcomes and process outcomes (use)

Restate study questions and summarize the answers suggested by the data, starting with primary outcomes and process outcomes (use).

subitem not at all important ○ 1 ○ 2 ○ 3 ● 4 ○ 5 essential

Clear selection

Does your paper address subitem 22-i? *

Copy and paste relevant sections from the manuscript (include quotes in quotation marks "like this" to indicate direct quotes from your manuscript), or elaborate on this item by providing additional information not in the ms, or briefly explain why the item is not applicable/relevant for your study

This feasibility RCT indicated that a larger-scale trial evaluating the effectiveness of DTx-MBI in youth with a concussion compared to an attention-matched sham intervention is viable, with four out of the five prespecified feasibility benchmarks–eligibility, recruitment, credibility, and retention–being met or exceeded. Only adherence fell slightly below the target threshold, with engagement levels comparable to other digital health interventions in youth [28], suggesting that minor adjustments could improve adherence in the large-scale efficacy RCT.

22-ii) Highlight unanswered new questions, suggest future research

Highlight unanswered new questions, suggest future research.

subitem not at all important ○ 1 ○ 2 ○ 3 ○ 4 ● 5 essential

Clear selection

Does your paper address subitem 22-ii?

Copy and paste relevant sections from the manuscript (include quotes in quotation marks "like this" to indicate direct quotes from your manuscript), or elaborate on this item by providing additional information not in the ms, or briefly explain why the item is not applicable/relevant for your study

These preliminary findings warrant further investigation in a fully powered trial to determine the intervention's efficacy and clarify its impact across recovery outcomes.

20) Trial limitations, addressing sources of potential bias, imprecision, and, if relevant, multiplicity of analyses

20-i) Typical limitations in ehealth trials

Typical limitations in ehealth trials: Participants in ehealth trials are rarely blinded. Ehealth trials often look at a multiplicity of outcomes, increasing risk for a Type I error. Discuss biases due to non-use of the intervention/usability issues, biases through informed consent procedures, unexpected events.

subitem not at all important ○ 1 ○ 2 ○ 3 ○ 4 ● 5 essential

Clear selection

Does your paper address subitem 20-i? *

Copy and paste relevant sections from the manuscript (include quotes in quotation marks "like this" to indicate direct quotes from your manuscript), or elaborate on this item by providing additional information not in the ms, or briefly explain why the item is not applicable/relevant for your study

Despite overall strong feasibility metrics, this study has some limitations. First, estimates of recruitment, eligibility, adherence, and retention were based on a modest sample size, which may limit precision. Second, the control group's treatment expectancy was qualitatively lower and just below the benchmark for being considered "good", which may suggest that some participants suspected they were not receiving the genuine cognitive training intervention, indicating partial unblinding or differential perceived value, despite the use of an active sham. These factors will need to be carefully addressed in the design and interpretation of the efficacy trial. Lastly, as expected for a feasibility study, it was not powered to detect efficacy, and outcome findings should be considered exploratory and interpreted accordingly.

21) Generalisability (external validity, applicability) of the trial findings

NPT: External validity of the trial findings according to the intervention, comparators, patients, and care providers or centers involved in the trial

21-i) Generalizability to other populations

Generalizability to other populations: In particular, discuss generalizability to a general Internet population, outside of a RCT setting, and general patient population, including applicability of the study results for other organizations

subitem not at all important ○ 1 ○ 2 ● 3 ○ 4 ○ 5 essential

Clear selection

Does your paper address subitem 21-i?

Copy and paste relevant sections from the manuscript (include quotes in quotation marks "like this" to indicate direct quotes from your manuscript), or elaborate on this item by providing additional information not in the ms, or briefly explain why the item is not applicable/relevant for your study

Not the goal of the study.

21-ii) Discuss if there were elements in the RCT that would be different in a routine application setting

Discuss if there were elements in the RCT that would be different in a routine application setting (e.g., prompts/reminders, more human involvement, training sessions or other co-interventions) and what impact the omission of these elements could have on use, adoption, or outcomes if the intervention is applied outside of a RCT setting.

subitem not at all important ○ 1 ○ 2 ● 3 ○ 4 ○ 5 essential

Clear selection

Does your paper address subitem 21-ii?

Copy and paste relevant sections from the manuscript (include quotes in quotation marks "like this" to indicate direct quotes from your manuscript), or elaborate on this item by providing additional information not in the ms, or briefly explain why the item is not applicable/relevant for your study

Not applicable for this study

OTHER INFORMATION

23) Registration number and name of trial registry

Does your paper address CONSORT subitem 23? *

Copy and paste relevant sections from the manuscript (include quotes in quotation marks "like this" to indicate direct quotes from your manuscript), or elaborate on this item by providing additional information not in the ms, or briefly explain why the item is not applicable/relevant for your study

Clinical trial.gov : NCT05105802

24) Where the full trial protocol can be accessed, if available

Does your paper address CONSORT subitem 24? *

Cite a Multimedia Appendix, other reference, or copy and paste relevant sections from the manuscript (include quotes in quotation marks "like this" to indicate direct quotes from your manuscript), or elaborate on this item by providing additional information not in the ms, or briefly explain why the item is not applicable/relevant for your study

The full protocol was published previously [45].

25) Sources of funding and other support (such as supply of drugs), role of funders

Does your paper address CONSORT subitem 25? *

Copy and paste relevant sections from the manuscript (include quotes in quotation marks "like this" to indicate direct quotes from your manuscript), or elaborate on this item by providing additional information not in the ms, or briefly explain why the item is not applicable/relevant for your study

This study was funded by a CHAMO Innovation grant (PI: AAL and RZ), a University of Ottawa Brain and Mind Research Institute team grant (PI: AAL), a Martin Osmond Research Grant Award at CHEO Research Institute (PI: AAL), and Ontario Brain Institute NERD Program (PI: AAL). VS is supported by a Canadian Institutes of Health Research postdoctoral fellowship.

X27) Conflicts of Interest (not a CONSORT item)

X27-i) State the relation of the study team towards the system being evaluated

In addition to the usual declaration of interests (financial or otherwise), also state the relation of the study team towards the system being evaluated, i.e., state if the authors/evaluators are distinct from or identical with the developers/sponsors of the intervention.

subitem not at all important ○ 1 ○ 2 ○ 3 ○ 4 ● 5 essential

Clear selection

Does your paper address subitem X27-i?

Copy and paste relevant sections from the manuscript (include quotes in quotation marks "like this" to indicate direct quotes from your manuscript), or elaborate on this item by providing additional information not in the ms, or briefly explain why the item is not applicable/relevant for your study

VS is supported by a Canadian Institutes of Health Research postdoctoral fellowship and receives royalties for the publication of a book on concussion by Flammarion. MC is one of the authors of the Mindfulness-Based Intervention for Concussion protocol used in this study, but she did not receive any financial benefit. She has received competitively-funded research grants from Canadian Institutes of Health Research (CIHR) and Michael Smith Health Research BC. RZ received financial support through competitively funded research grants from the Canadian Institutes of Health Research, Ontario Neurotrauma Foundation, Physician Services Incorporated Foundation, Children's Hospital of Eastern Ontario Foundation, University of Ottawa Brain and Mind Research Institute, Ontario Brain Institute, National Football League, Ontario Ministry of Health, Public Health Agency of Canada, Health Canada, Parachute Canada, and Ontario SPOR Support Unit. RZ was supported by a Tier 1 Clinical Research Chair in Pediatric Concussion from the University of Ottawa. All grant funding goes directly to the institution. RZ sits on the board of directors for the North American Brain Injury Society, which is a volunteer (unpaid) role. RZ is a founding partner and a minority shareholder of 360 Concussion Care (a learning health system and network of interdisciplinary concussion clinics in Ontario); no proceeds have been transferred to RZ. NR holds a Canada Research Chair (Tier 2) in Pediatric Concussion and has received research funding from the Canadian Institutes of Health Research (CIHR), Social Sciences and Humanities Research Council (SSHRC), Ontario Neurotrauma Foundation (ONF), Public Health Agency of Canada (PHAC), Parachute Canada, Special Olympics Canada, Greater Toronto Hockey League, Dr. Tom Pashby Sport Safety Fund, Holland Bloorview Kids Rehabilitation Hospital and Scotiabank. NR is an Executive Board Member for the International Pediatric Brain Injury Society (IPIBS) which is an unpaid and volunteer role. All other authors declare no other conflicts of interest. BJS is the Chief Scientist and CEO of Mobio Interactive PTE LTD (Singapore), the parent company of Mobio Interactive Inc. (Toronto, Canada), and he owned approximately 22% of the company at the time of manuscript submission. BJS exclusively served as a technical liaison for the study and did not contribute to the specific study design, or select the outcome measures, nor did he directly contribute to, or have influence over, data collection or analysis. AAL is one of the authors of the Mindfulness-Based Intervention for Concussion protocol used in this study, but she did not receive any financial benefit. Andrée-Anne Ledoux has received competitively-funded research grants from Canadian Institutes of Health Research (CIHR), Ontario Neurotrauma Foundation (ONF), Physician Services Incorporated (PSI) Foundation, CHEO Foundation, Ontario Brain Institute (OBI), University of Ottawa Brain and Mind Research. AAL received funding from the Ontario Brain Institute Neurotech Early Research & Development Program for a separate component of the study. AAL has no other conflict of interest to disclose.

About the CONSORT EHEALTH checklist

As a result of using this checklist, did you make changes in your manuscript? *

- ○ yes, major changes
- ○ yes, minor changes
- ● no

What were the most important changes you made as a result of using this checklist?

No changes were necessary

How much time did you spend on going through the checklist INCLUDING making changes in your manuscript *

2 hours. The system is really buggy.

As a result of using this checklist, do you think your manuscript has improved? *

- ○ yes
- ◉ no
- ○ Other: __________

Would you like to become involved in the CONSORT EHEALTH group?

This would involve for example becoming involved in participating in a workshop and writing an "Explanation and Elaboration" document

- ○ yes
- ◉ no
- ○ Other: __________

Clear selection

Any other comments or questions on CONSORT EHEALTH

Your answer

STOP - Save this form as PDF before you click submit

To generate a record that you filled in this form, we recommend to generate a PDF of this page (on a Mac, simply select "print" and then select "print as PDF") before you submit it.

When you submit your (revised) paper to JMIR, please upload the PDF as supplementary file.

Don't worry if some text in the textboxes is cut off, as we still have the complete information in our database. Thank you!

Final step: Click submit !

Click submit so we have your answers in our database!

Submit   Clear form

Never submit passwords through Google Forms.

This content is neither created nor endorsed by Google. - Contact form owner - Terms of Service - Privacy Policy

Does this form look suspicious? Report
